# Unveiling the distribution of free and bound phenolic acids, flavonoids, anthocyanins, and proanthocyanidins in pigmented and non-pigmented rice genotypes

**DOI:** 10.3389/fpls.2024.1324825

**Published:** 2024-04-10

**Authors:** Gosangi Avinash, Neerja Sharma, Kalluri Rajendra Prasad, Rupinder Kaur, Gurjeet Singh, Nagaraju Pagidipala, Thiyagarajan Thulasinathan

**Affiliations:** ^1^ Department of Biochemistry, Punjab Agricultural University, Ludhiana, India; ^2^ Department of Plant Breeding and Genetics, Punjab Agricultural University, Ludhiana, India; ^3^ Department of Genetics and Plant Breeding, Professor Jayashankar Telangana State Agricultural University, Hyderabad, Telangana, India; ^4^ Indian Institute of Rice Research, Indian Council of Agricultural Research (ICAR), Hyderabad, Andhra Pradesh, India; ^5^ Tamil Nadu Agricultural University, Coimbatore, Tamil Nadu, India

**Keywords:** pigmented and non-pigmented rice, phenolic acids, flavonoids, anthocyanins, proanthocyanidins, antioxidant activity

## Abstract

The total phenolic content, phenolic acid profile, anthocyanins, proanthocyanidins, flavonoids, and antioxidant capacity of the whole-grain and bran portion of sixteen distinct rice genotypes that correspond to three distinct pericarp bran colors—black, red, and non-pigmented (NP)—were examined. Ten free and bound phenolic acids, as well as two flavonoids, were analyzed using HPLC-PDA. The flavonoids included kaempferol and catechin hydrate, and the free phenolic acids included gallic acid, 2,5-dihydroxybenzoic acid, vanillic acid, syringic acid, p-coumaric acid, chlorogenic acid, trans-cinnamic acid, trans-ferulic acid, p-coumaric acid, and sinapic acid. Trans-ferulic acid (207.39 mg/kg), p-hydroxybenzoic acid (94.36 mg/kg), and p-coumaric acid (59.75 mg/kg) were the principal bound phenolic acids in pigmented rice genotypes, whereas in NP genotypes they were trans-ferulic acid (95.61 mg/kg) and p-hydroxybenzoic acid (58.32 mg/kg). The main free phenolic acid was syringic acid (120.43 mg/kg) in all genotypes. 2,5-dihydroxybenzoic acid was also detected in NP genotypes, mainly in the bound form (4.88 mg/kg). NP genotypes Basmati 386 and Punjab Basmati 7 also displayed high content of bran flavonoids (1001 and 1028 mg CE/100 g). The bound form of phenolics had significant DPPH and ABTS + activity. This study found wide diversity in the phenolic acid profile, total phenolic constituents, and antioxidant activity in the bran and whole grain of pigmented and NP rice. The individual phenolic acids in free and bound forms in different fractions of the grain were found to exert their antioxidant activity differently. The results obtained will provide new opportunities to improve the nutritional quality of rice with enhanced levels of phytochemicals in the ongoing breeding programs. Black rice bran contains a high level of phytochemicals and thus has a potent pharmaceutical role. This information would enhance the use of whole-grain and bran of pigmented rice in food product development by food technologists. Further studies may be focused on clinical trials with respect to cancer and diabetes.

## Introduction

For more than half of the world’s population, rice is a staple part of the diet. Approximately 90% of the world’s rice is produced and consumed in Asian nations, where it is the primary source of nutrient-dense energy. Pigmented rice cultivars, including purple, black, and red rice, have garnered significant attention in recent years due to their high bioactive chemical content, antioxidant properties, anti-inflammatory properties, and other health advantages (Alves et al., 2016). Consuming rice grains with phytochemicals related to antioxidant activity may lessen the incidence of chronic illnesses (Sharmila et al., 2023). In India, numerous colored rice varieties possessing various therapeutic characteristics are being investigated (Siva et al., 2013). Because of their antioxidant qualities, secondary metabolites found in colored rice contribute to the prevention of lifestyle diseases such as cancer, heart disease, and aging-related illnesses (Shen et al., 2009). Pericarp coloration in rice grains is primarily caused by the type and concentration of polyphenols, which vary among genotypes. Furthermore, hypoallergenic protein anchors and several functional chemicals, such as polyphenolic compounds, are present in the embryo fraction and bran layer of rice grains (Iqbal et al., 2005). The accumulation of numerous phenolic chemicals, which are stored in the vacuole and include anthocyanins, phenolic acids, and flavonoids, is positively connected with the bran layers of colored rice (Sumczynski et al., 2016; Shao et al., 2018). Phenolic acids are typically the main hydrophilic antioxidants found in white rice, whereas proanthocyanidins and anthocyanins are found in red and black rice, respectively (Finocchiaro et al., 2010; Zaupa et al., 2015). Black rice has free-form anthocyanin accumulation in the outer layers, primarily cyanidin-3-O-glucoside and peonidin-3-O-glucoside (Shao et al., 2014). Both soluble and bound forms of phenolic constituents are found in rice; in light brown-colored rice grains, the soluble form makes up 38% to 60% (Adom and Liu, 2002; Mira et al., 2009) of the total polyphenolic content, whereas in black and red pericarp-colored rice grains, about 81% of bound phenolics are present (Mira et al., 2009). A significant amount of free phenolic acids, namely ferulic acid (FA) and p-coumaric acid (p-CA), are incorporated into lignin and cell wall polysaccharides (arabinoxylans) to form insoluble phenolics (Zhou et al., 2004).

The main free phenolic acids found in black rice are ferulic acid (FA), trans-p-coumaric acid (t-CA), and protocatechuic acid (PCA); bound forms of these phenolic acids are found in Aquercetin and vanillic acid (VA) (Shao et al., 2018; Kunnam et al., 2023). When it comes to the various forms of phenolic compounds and their respective uses, the soluble form, which is easily absorbed in the stomach and small intestine, has the ability to stop free radicals from oxidizing biological macromolecules (Shao et al., 2015). On the other hand, the bound forms are absorbed in the intestine only after being broken down by hydrolytic enzymes, and some of them are released into the colon by the colonic microflora (Saura et al., 2007). According to reports, the main antioxidants in rice are polyphenols, which include phenolic acids, anthocyanins, and proanthocyanidins (Min et al., 2011; Bani et al., 2023).

The distribution of phenolic acids exhibits varietal differences, and rice bran has the highest total phenolic content (TPC) among four different fractions of whole rice grain (Shao et al., 2014; Ti et al., 2014). Overall, FA*, p*-CA, syringic (SYA) VA, sinapic (SIA) caffeic (CA) *p*-hydroxybenzoic (*P-*HBA), and protocatechuic acid are present in the whole rice grain, of which ferulic acid is the most abundant phenolic acid (Zhang et al., 2015), in insoluble-bound fraction. The phenolic contents were positively correlated with the antioxidant capacity (Paiva et al., 2016). Identifying and characterizing bioactive substances is one of the most popular topics in rice nutritional quality research right now. However, the differences in bound and free phenolics and their relation to antioxidant activity have not been well understood, partly due to genotypic diversity.

Detailed investigations of phenolic constituents and their relation to antioxidant activities may promote the development of rice-based functional foods. Moreover, rice varieties that are grown and consumed throughout the world mostly possess light brown pericarp. Keeping in view the health benefits of colored rice, the present study aimed to carry out a comparative evaluation of phenolic acid profiles of free and bound forms in bran and whole grain and antioxidative abilities of black, red, and NP pericarp rice to generate a database for rice breeders to develop rice varieties rich in polyphenolic constituents.

## Materials and methods

### Rice samples

Sixteen rice (*Oryza sativa* L.) genotypes, which consisted of seven non-pigmented light brown pericarps rices viz; PR114, PR121, PR126, Bas370, Bas386, Pb7, and Pb1121; three black rice genotypes, Farmer Collection (Punjab), Farmer Collection (Arunachal Pradesh), Chakhao poireiton; and six red rice genotypes viz; Ghaselu, Dumai, Kammra, Bakung, Maintimolosty, and Yangkum, were selected for the study and cultivated during the wet season (*Kharif*) of the year 2020 (**Table 1**) in the research field of Punjab Agricultural University, Ludhiana India. After maturing, the grains were harvested, sun-dried to a moisture content of about 13%, stored in air-tight plastic bags at room temperature for three months, and then stored at 4°C in the dark till analysis. The rough rice samples were de-hulled on testing Husker (THU 35 B (3) -T, Satake (Thailand) Co. Ltd.) and milled using a rice polisher (Model 60-220-50 DT, Grainman Machinery Mfg. Corp., Miami, FL) and graded on a Satake Rice Machine to yield head rice. The head rice was powdered with a cyclotec mill fitted with an 150-micron sieve.

**Table 1:** Designations of rice genotypes used in the present study.

S. No.	Designation	(IRGC) No.	Abbreviation
BLACK RICE			
1.	Farmer collection (Punjab)	–	FCP
2.	Farmer collection (Arunachal Pradesh)	–	FCAP
3.	Chakhao poireiton	–	CP
RED RICE			
4.	Ghaselu	121344	GSL
5.	Dumai	121198	DMI
6.	Kammra	121377	KMA
7.	Bakung	121193	BKG
8.	Maintimolosty	121791	MSTY
9.	Yangkum	117593	YKM
NON-PIGMENTED RICE			
10.	PR I14	–	PR 114
11.	PR I21	–	PR I21
12.	PR I26	–	PR I26
13.	Basmati 370	–	Bas 370
14.	Basmati 386	–	Bas 386
15.	Punjab Basmati 7	–	Pb Bas 7
16.	Pusa Basmati 1121	–	PB 1121

### Chemicals and reagents

2,2′-Azino-bis-(3-ethylbenzothiazoline-6-sulfonic acid) diammonium salt (ABTS.+), 2,2 diphenyl-1-picrylhydrazyl (DPPH), 6-hydroxy-2,5,7,8-tetramethyl chroman-2-carboxylic acid (Trolox), analytical standards of phenolic acids *viz*., gallic acid; GA, 2,5-dihydroxybenzoic acid; 2,5-DHA*, p*-hydroxybenzoic acid; *p*-HA, vanillic acid; VA, syringic acid; SYA, *p*- coumaric acid; *p*-CA, chlorogenic acid; CHL, *t*-cinnamic acid; *t*-ferulic acid; *t*-FA, Sinapic acid; SIA, and the flavonoids kaempferol; KAM, (±) and Catechin hydrate CH were purchased from Sigma Aldrich, Chemical Co. (St. Louis, MO, USA). HPLC grade methanol, acetic acid, and acetonitrile used for extraction and HPLC analysis were purchased from Merck, India Pvt. Ltd. All other chemicals used were of analytical grade or above.

### Extraction of free and bound phenolic constituents

Extraction of free phenolics was accomplished based on the method reported by Shao et al. (2014) with little modification. Defatted rice flour (1.0 g) was extracted twice with 20mL of 80% methanol. Each time, the mixture was kept on a mechanical shaker (multi-speed oscillator HY-8, Changzhou Guohua Electric Appliance Co., Ltd, Jiangsu, China) and subsequently centrifuged (TGL-20B highspeed desktop centrifuge, Shanghai Anting Scientific Instrument Factory, Shanghai, China) at 4000g for 20 min at room temperature. The supernatants were pooled after adjusting the pH to 1.5-2.0 and were concentrated using a rotary evaporator (IKA RV10 digital V, Staufen, Germany). The concentrated free fraction was extracted three times using ethyl acetate (20mL each time). The ethyl acetate extracts were pooled and rotary evaporated. The dried extracts were dissolved in 50% methanol (5 mL) and used as crude-free phenolic extracts. The solid residue after the extraction of free phenolics was washed with distilled water and then digested with 4 M NaOH (20 mL) at room temperature on a shaker (multi-speed oscillator-8, Changzhou Guohua Electric Appliance Co., Ltd, Jiangsu, China) for 2hr. The mixture was then adjusted to pH 1.5-2.0 with concentrated HCl and extracted thrice with 60 mL of ethyl acetate. The combined ethyl acetate fractions were concentrated and dissolved in 5 mL of 50% methanol. The extracts were used as bound phenolic extracts.

### Determination of antioxidant activity

The spectrophotometric assay of DPPH and ABTS + radical scavenging activity was done according to Shao et al. (2014). In brief, 100 μmol/L of DPPH radical solution was prepared in methanol. The appropriately diluted crude extract (100 μL) was added to1.5 mL DPPH solution. After 30 min of incubation at room temperature in the dark, the absorbance at 517 nm was measured. The DPPH radical scavenging activity of the sample was measured as percentage inhibition of absorbance. Each extract was measured in triplicate. The total ABTS + radical cation method was carried out as described by Shen et al. (2009). ABTS + radical cation was generated by reacting 7 mM ABTS + and 2.45 mM potassium persulfate at room temperature in the dark for 16 h. The ABTS+ solution was diluted with 80% ethanol to an absorbance of around 0.7 at 734 nm. ABTS + solution (3.9 mL, absorbance of 0.7) was added to 0.1 mL of the extract and mixed thoroughly. The reaction mixture was kept at room temperature for 6 min, and the absorbance was immediately recorded at 734 nm. The Trolox standard solution in 80% ethanol was prepared and assayed under the same conditions. Results were expressed as μM Trolox equivalent antioxidant capacity per gram dry weight (μM TEAC/g dry wt.).

### Determination of anthocyanin content

Anthocyanin content was estimated according to the method described by Pal et al. (2019). First, 1 g of rice powder was homogenized with 5 mL acidified organic solvent (95% methanol: 1.5 N HCl, 85:15, pH = 1) and kept overnight. Centrifugation was then carried out at 200C for 10 min at 9200g. The final volume of supernatant was 10 mL, and its absorbance was measured at 535 nm. The total anthocyanin content of the sample was expressed as mg of cyanidin-3-glucoside per 100 g of sample and calculated according to Alessandra et al. (2016).

### Determination of total proanthocyanidin content

Each 1.0 g sample was extracted with 80% methanol using a shaker (Shaking Heidolph Unimax 1010DT, Schwabach, Germany) at 300 rpm for 8 h, and then centrifuged at 12000 g for 20 min. Total proanthocyanidin content was measured using the vanillin assay method (Sun et al., 1998) with little modification. The extract solution (0.4 mL) was mixed with 1 mL of 9M sulphuric acid and 1 mL of 1% vanillin in methanol (w/v). A control mixture of the sample was prepared by adding 100% methanol instead of the vanillin solution for correcting the absorbance by non-vanillin reactive compounds to eliminate the influence of the interference (e.g., anthocyanins). After incubation for 15 min in a 30°C water bath, the absorbance of the sample and control mixtures was measured at 500 nm against a reagent blank and their difference was used to determine the total proanthocyanidin of the samples, which was expressed as mg catechin (CE)/g sample (dry basis).

### Determination of total flavonoids content

The total flavonoid content was determined using a modified spectrophotometric method described by (Dewanto et al., 2002). A 0.3 mL aliquot of the extract used for the estimation of proanthocyanidin was added to a tube containing1.5 mL of distilled water. The extract was then mixed with 0.09 mL 5% NaNO2 solution for 6 min followed by 0.18 mL 10% AlCl3.6H2O solution for 5 min. The mixture was then added to 0.6 mL 1 M NaOH and distilled water was added to make the total volume 3 mL. The absorbance was determined at 510 nm. Total flavonoid content was calculated from a standard curve of (+)-catechin (10-100µg) and expressed as mg (+)-catechin equivalents (CE) per 100 g DW of the sample.

### HPLC analysis of phenolic acids

**Figure 1:**
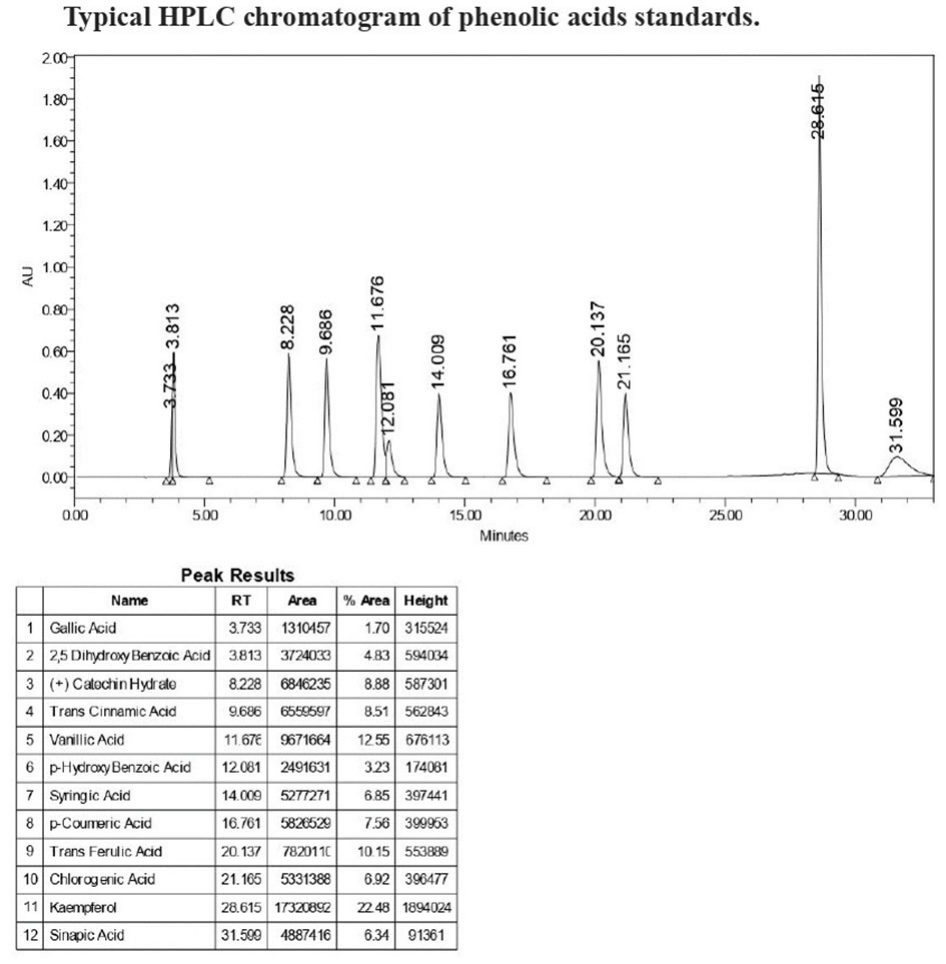
Typhical HPLC chromatogram of phenolic acids standards.

The phenolic acid content was analyzed by an HPLC system that consisted of a binary pump (Waters 2695), an autosampler (Waters 2707), and a PDA detector (Waters W2998). A C18 column of dimensions 250 mm 4.6 mm with a 5 µm pore size (XBridge, Waters) was used for separation. The mobile phase consisted of A (87:3:10 Water: Acetonitrile (ACN): Formic acid) and B (40:50:1 Water: ACN: Formic acid). The flow rate was 1 mL/min. A 33 min gradient was set according to the method of Shao et al. (2014) with some modifications. The injection volume was 10 µL. The column temperature was kept at 33C. Free and bound phenolic extracts (section 2.3) were filtered through 0.45 µm membrane filters before analysis. The phenolic acids were detected at wavelengths of 280nm and quantified using the external calibration curves according to the retention time of phenolic acid standards (**Figure 1**).

### Statistical analysis

All the analyses were carried out at least in triplicate, and the results were reported as means ± standard deviation (SD). Analysis of variance (ANOVA) was used to analyze the data by using a completely randomized design (CRD), using SAS version 9.4 software. Tukey’s *post-hoc* test was performed to compare the difference of mean of samples.

## Results

### Whole-grain phenolic acids

Phenolic acid content of ten phenolic acids and two flavonoids were estimated in the free and bound form in bran and whole grain in sixteen rice genotypes: three black, six red, and seven NP. Phenolic acids exist in free and bound forms and studies have shown that two groups of phenolic acids are present in rice grain (Irakli et al., 2012), *viz.*, derivatives of hydroxybenzoic acids (gallic acid, GA; 2,5-dihydroxybenzoic acid, 2,5-DHA*; p*- hydroxybenzoic acid, *p*-HBA; vanillic acid, VA; syringic acid, SYA) and derivates of hydroxycinnamic acids (chlorogenic acid, CHA; trans-cinnamic acid, *trans*-CA; *trans*-ferulic acid, *trans*-FA; p-coumaric acid, *p*-CA; and sinapic acid, SIA). Flavonoids such as Kaempferol (KAF) (Bagchi et al., 2021) and (±) Catechin hydrate (CH) (Das et al., 2016) have also been reported. GA existed in both the free and bound form in black, red, and NP rice genotypes, with its content ranging from 2.69 to 4.59 and 4.03 to 4.51; 2.62 to 4.91 and 1.93 to 8.56; and 1.55 to 6.32 and 0.1 to 2.68mg/kg, respectively (**Tables 2**, **3**). Detectable content of GA in whole grain NP rice genotype Bas 386 in bound form was not found. 2,5-DHA existed in both the free and bound form in black, red, and NP rice genotypes, with its content ranging from 0.09 to 0.26 and 2.01 to 12.42; 0.08 to 0.57 and 9.01 to 14.73; and 0.07 to 0.29 and 0.4 to 16.15mg/kg, respectively. There was no detectable 2,5-DHA in the free form in red rice genotype DMI and NP rice genotype Bas 370, Pb Bas 7, and Bas 386. VA existed in both the free and bound form in black, red, and NP rice genotypes, with its content ranging from 0.2-6.09 and 0.012 to 0.015; 0.34 to 5.89 and 0.02 to 0.8; and 0.29 to 5.3 and 0.03 to 0.42 mg/kg, respectively. *p*-HBA existed in both the free and bound form in black, red, and NP rice genotypes with its content ranging from 24.67 to 30.96 and 161.61 to 210.46; 3.49 to 41.81 and 0.02 to 197.74; and 4.41 to 36.69 and 2.24 to 114.22 mg/kg, respectively. SYA existed in both the free and bound form in black, red, and NP rice genotypes, with its content ranging from 18.81-261.19 and 5.06-6.99; 0.75-156.6 and 1.5-7.42; and 2.95-182.39 and 0.04-2.75 mg/kg, respectively. CHA existed in both the free and bound form in black, red, and NP rice genotypes, with its content ranging from 1.21-7.73 and 4.31-5.24; 0.36-5.15 and 1.99-7.85; and 0.34-3.22 and 0.12-5.43 mg/kg, respectively. *Trans*-CA existed in both the free and bound form in black, red, and NP rice genotypes, with its content ranging from 0.23-1.55 and 0.86-2.77; 0.24-3.52and 0.85-2.67; and 0.23-3.51 and 0.04-2.18 mg/kg, respectively. *trans*-FA existed in both free and bound form in black, red, and NP rice genotypes, with its content ranging from 1.47-5.32 and 229.06-239.35; 1.40-5.78 and 158.6.-230.34; and 1.47-3.65 and 10.6-182.38 mg/kg, respectively. *p*-CA existed in both the free and bound form in black, red, and NP rice genotypes, with its content ranging from 0.72-2.1 8and 35.21-49.65; 0.69-2.22 and 38.49-98.44; and 0.61-2.37 and 5.77-75.54 mg/kg, respectively. SIA existed in both the free and bound form in black, red, and white rice genotypes, with its content ranging from 0.83-16.14 and 2.73-5.61; 0.79-21.26 and 1.65-2.79; and 0.67-127.72and 0.65-5.17 mg/kg, respectively. The flavonoid KAF existed in both the free and bound form in black, red, and NP rice genotypes, with its content ranging from 1.92-45.9 and 1.23-1.48; 19.04-94.85 and 1.13-1.95; and 0.53-156.82 and 0.56-38.11mg/kg, respectively.CH existed in both the free and bound form in black, red, and NP rice genotypes, with its content ranging from 5.06-17.68 and 26.35 - 35.73; 4.78-20.01 and 21.94-82.29; and 2.15-13.82 and 6.07-33.68 mg/kg, respectively.

**Table 2:** The content of free phenolic acids in the whole grain.

Genotypes	Hydroxybenzoic acid					Hydroxycinnamic acid					Flavonoids		
Black rice	GA	2,5-DHA	VA	*p*-HBA	SYA	CHA	*t*-CA	*t*-FA	*p*-CA	SIA	KAF	CH	TOTAL
**FCP**	2.69c	0.26a	1.1b	30.96a	147.66b	5.08b	0.23c	3.84b	1.54b	16.14a	45.9a	5.06c	260.46b
**FCAP**	4.59a	0.23b	0.2c	30.29a	267.19a	7.71a	0.93b	5.32a	2.18a	1.91b	1.92c	10.94b	333.44a
**CP**	2.92b	0.09c	6.09a	24.67b	18.81c	1.21c	1.55a	1.47c	0.72c	0.83c	19.04b	17.68a	95.09c
**Mean**	**3.40^A^**	**0.19^A^**	**2.47^A^**	**28.64^A^**	**144.55^A^**	**4.66^A^**	**0.9^B^**	**3.55^A^**	**1.48^A^**	**6.29^B^**	**22.29^B^**	**11.23^A^**	**229.65^A^**
**Range**	**2.69-4.59**	**0.09-0.26**	**0.2-6.09**	**24.67-30.96**	**18.81-261.19**	**1.21-7.73**	**0.23-1.55**	**1.47-5.32**	**0.72-2.18**	**0.83-16.14**	**1.92-45.9**	**5.06-17.68**	**95.09-333.44**
**P-Value**	**<0.001**	**<0.001**	**<0.001**	**<0.001**	**<0.001**	**<0.001**	**<0.001**	**<0.001**	**<0.001**	**<0.001**	**<0.001**	**<0.001**	**<0.001**
Red rice													
**GSL**	2.87d	0.45b	0.34d	7.19e	3.82c	0.45de	2.53c	3.79d	1.11d	17.58b	94.85a	5.45d	140.43c
**DMI**	4.91a	n.d	1.13c	41.81a	156.17a	4.76b	0.24e	3.42e	2.22a	15.87c	83.67b	5.14e	319.36a
**KMA**	3.2c	0.42c	0.39d	8.29d	4.46c	0.58d	2.96b	4.91b	1.06d	21.26a	25.65d	7.7c	80.89e
**BKG**	2.83d	0.08e	5.89a	24.56c	17.8b	1.29c	1.49d	1.40f	0.69e	0.79e	18.06e	20.01a	94.89d
**MSTY**	3.78b	0.57a	1.48b	3.49f	0.75c	0.36e	3.52a	5.78a	1.35c	0.92e	24.81d	10.07b	56.87f
**YKM**	2.62e	0.24d	1c	32.59b	156.6a	5.15a	n.d	4.21c	1.66b	15d	42.31c	4.78f	266.15b
**Mean**	**3.36^A^**	**0.29^AB^**	**1.70^B^**	**19.65^B^**	**56.6^B^**	**2.09^B^**	**1.79^A^**	**3.91^A^**	**1.34^AB^**	**11.90^AB^**	**48.22^A^**	**8.85^B^**	**159.76^C^**
**Range**	**2.62-4.91**	**0-0.57**	**0.34-5.89**	**3.49-41.81**	**0.75-156.6**	**0.36-5.15**	**0-3.52**	**1.40-5.78**	**0.69-2.22**	**0.79-21.26**	**19.04-94.85**	**4.78-20.01**	**56.87-319.36**
**P-Value**	**<0.001**	**<0.001**	**<0.001**	**<0.001**	**<0.001**	**<0.001**	**<0.001**	**<0.001**	**<0.001**	**<0.001**	**<0.001**	**<0.001**	**<0.001**
Non- pigmented rice													
**PR 114**	2.29de	0.07d	0.3e	4.41f	2.95e	0.34e	2.01c	2.41b	0.91c	127.72a	156.82a	5.07c	305.3a
**PR 121**	2.38d	0.08c	5.3a	20.58c	13.3c	1.1c	1.31d	1.68c	0.93c	0.71d	9.25d	13.72a	70.33f
**PR 126**	2.24e	0.13b	0.48d	32.19b	182.39a	3.22a	0.27f	3.61a	2.37a	62.38b	0.53e	4.78d	294.58b
**BAS 370**	6.32a	n.d	0.29e	7.79e	3.55de	0.36e	2.54b	3.65a	1.11b	15.18c	72.07b	6.02b	118.88e
**BAS 386**	1.55f	n.d	2.83c	16.03d	166.84b	1.61b	0.23f	1.53d	0.82d	16.73c	1.05e	2.15e	211.37c
**Pb Bas7**	5.54b	n.d	4.39b	36.69a	7.48d	n.d	3.51a	n.d	n.d	16.02c	62.4c	13.82a	149.85d
**PB 1121**	2.55c	0.29a	5.3a	20.58c	12.49c	0.92d	1.16e	1.47d	0.61e	0.67d	9.41d	13.72a	69.16f
**Mean**	**3.27^B^**	**0.08^B^**	**2.7^A^**	**19.75^B^**	**55.57^B^**	**1.08^B^**	**1.58^A^**	**2.05^B^**	**0.96^B^**	**34.2^A^**	**44.5^AB^**	**8.47^B^**	**174.21^B^**
**Range**	**1.55-6.32**	**0-0.29**	**0.29-5.3**	**4.41-36.69**	**2.95-182.39**	**0-3.22**	**0.23-3.51**	**0-3.65**	**0-2.37**	**0.67-127.72**	**0.53-156.82**	**2.15-13.82**	**69.16-305.3**
**P-Value**	**<0.001**	**<0.001**	**<0.001**	**<0.001**	**<0.001**	**<0.001**	**<0.001**	**<0.001**	**<0.001**	**<0.001**	**<0.001**	**<0.001**	**<0.001**
**Average (Overall)**	**1.55**	**0**	**0.2**	**3.49**	**0.75**	**0**	**0**	**0**	**0**	**0.67**	**0.53**	**2.15**	**56.87**
**Min (Overall)**	**6.32**	**0.57**	**6.09**	**41.81**	**267.19**	**7.73**	**3.52**	**5.78**	**2.37**	**127.72**	**156.82**	**17.68**	**333.44**
**Max (Overall)**	**4.78**	**0.57**	**5.89**	**38.32**	**266.44**	**7.73**	**3.52**	**5.78**	**2.37**	**127.05**	**156.3**	**15.54**	**276.57**

Each value represents the mean ± SD of three independent replications.

The results are presented as mg/kg rice flour, and values in each column with different capital letters are significantly different (P < 0.001) means of the three colored groups, while the different small letters in each column are significantly different (P < 0.001) content of phenolic acids in each colored group. GA, gallic acid: 2,5-DHA, 2,5-dihydroxybenzoic acid; VA, vanillic acid; p- HBA, p-hydroxybenzoic acid; SYA, syringic acid; CHA, Chlorogenic acid; t-CA, trans-cinnamic acid; t-FA, trans-ferulic acid; p-CA, p-coumaric acid; SIA, sinapic acid; KAF, kaempferol; CH, catechin hydrate. n.d: not detected.

Bold values represent the mean, range, and p-value of individual phenolic acids.

**Table 3:** The content of bound phenolic acids in the whole grain.

Genotypes	Hydroxybenzoic acid					Hydroxycinnamic acid					Flavonoids		
Black rice	GA	2,5 -DHA	VA	*p*-HBA	SYA	CHA	*t*-CA	-FA	*p*-CA	SIA	KAF	CH	TOTAL
**FCP**	4.51a	2.01c	0.013b	210.46a	6.99a	5.24a	2.77a	239.35	46.96b	3.00b	1.48a	33.37b	556.15a
**FCAP**	4.48a	12.42a	0.015a	161.61c	5.09b	4.31c	2.49b	233.87	49.65a	5.61a	1.3b	26.35c	507.26b
**CP**	4.03b	9.89b	0.012c	195.45b	5.06b	4.86b	0.86c	229.06	35.21c	2.73b	1.23c	35.73a	524.1b
**Mean**	**4.35^A^**	**8.11^B^**	**0.013^B^**	**189.18^A^**	**5.71^A^**	**4.8^A^**	**2.04^A^**	**234.09^A^**	**43.94^A^**	**3.78^A^**	**1.34^B^**	**31.82^B^**	**529.17^A^**
**Range**	**4.03-4.51**	**2.01-12.42**	**0.012-.0.015**	**161.61-210.46**	**5.06-6.99**	**4.31-5.24**	**0.86-2.77**	**229.06-239.35**	**35.21-49.65**	**2.73-5.61**	**1.23-1.48**	**26.35-35.73**	**507.26-556.15**
**P-Value**	**<0.001**	**<0.001**	**<0.001**	**<0.001**	**<0.001**	**<0.001**	**<0.001**	**NS**	**<0.001**	**<0.001**	**<0.001**	**<0.001**	**<0.001**
Red rice													
**GSL**	3.88b	14.08ab	0.02f	197.74a	7.42a	4.79e	2.67a	226.83a	40.98cd	2.79a	1.45b	30.73d	533.39a
**DMI**	2.05e	14.73a	0.07e	0.02d	3.52b	5.42c	1.27c	202.72b	39.28de	1.77d	1.16c	30.48d	302.48de
**KMA**	2.58d	13.54b	0.15c	5.85cd	1.52d	5.74b	1.08d	191.81b	38.49e	2.04c	1.13c	48.08c	312.02d
**BKG**	1.93e	12.05c	0.11d	4.48cd	2.89c	5.14d	0.85e	194.59b	42.08c	1.65e	1.17c	21.94e	288.87e
**MSTY**	2.96c	10.7d	0.34b	9.45c	2.71c	7.85a	2.38b	230.34a	98.44a	1.76d	1.95a	82.29a	451.35c
**YKM**	8.56a	9.01cd	0.8a	129.28b	1.9d	1.99f	1.01c	158.6c	69.52b	2.44b	1.44b	49.10b	433.56 b
**Mean**	**3.66^A^**	**12.74^A^**	**0.24^A^**	**57.80^B^**	**3.32^B^**	**5.15^A^**	**1.54^AB^**	**200.8^A^**	**54.7^A^**	**2.07^B^**	**1.38^B^**	**43.77^A^**	**386.94^B^**
**Range**	**1.93-8.56**	**9.01-14.73**	**0.02-0.8**	**0.02-197.74**	**1.5-7.42**	**1.99-7.85**	**0.85-2.67**	**158.6-230.34**	**38.49-98.44**	**1.65-2.79**	**1.13-1.95**	**21.94-82.29**	**288.87-533.39**
**P-Value**	**<0.001**	**<0.001**	**<0.001**	**<0.001**	**<0.001**	**<0.001**	**<0.001**	**<0.001**	**<0.001**	**<0.001**	**<0.001**	**<0.001**	**<0.001**
Non- pigmented rice													
**PR 114**	2.68a	10.21b	0.42a	107.62b	0.04f	1.51d	2.18a	16.16f	75.54a	0.65f	1.49d	26.78b	245.27b
**PR 121**	2.62a	16.15a	0.21d	4.32e	0.63c	5.43a	1.21c	182.38a	34.48b	1.33d	1.00d	33.68a	283.43a
**PR 126**	0.1d	0.49e	0.03f	40.21d	0.43de	0.12f	1.67b	26.7d	5.77f	2.21c	12.73c	6.07f	96.52f
**BAS 370**	0.8c	3.26c	0.25c	114.22a	2.75a	1.66c	1.03d	10.6g	11.39de	5.17a	0.79d	17.79c	169.71d
**BAS 386**	n.d	3.16c	0.11e	80.8c	2.33b	0.66e	0.04f	83.44b	9.91e	1.15e	0.56d	12.1d	194.24c
**Pb Bas 7**	0.79c	0.4e	0.31b	2.24e	0.46d	1.61c	0.72e	21.93e	12.7d	3.16b	15.11b	11.72d	71.15g
**PB 1121**	1.08b	1.97d	0.11e	2.78e	0.36e	2.65b	1.09d	37.73c	18.16c	3.13b	38.11a	10.52e	117.71e
**Mean**	**1.15^B^**	**5.09^B^**	**0.2^A^**	**50.31^B^**	**1.00^C^**	**1.95^B^**	**1.13^B^**	**54.13^B^**	**23.99^B^**	**2.4^B^**	**9.97^A^**	**16.95^C^**	**168.29^C^**
**Range**	**0-2.68**	**0.4-16.15**	**0.03-0.42**	**2.24-114.22**	**0.04-2.75**	**0.12-5.43**	**0.04-2.18**	**10.6-182.38**	**5.77-75.54**	**0.65-5.17**	**0.56-38.11**	**6.07-33.68**	**71.15-283.43**
**P-Value**	**<0.001**	**<0.001**	**<0.001**	**<0.001**	**<0.001**	**<0.001**	**<0.001**	**<0.001**	**<0.001**	**<0.001**	**<0.001**	**<0.001**	**<0.001**
**Average (Overall)**	**2.76**	**8.52**	**0.18**	**79.3**	**2.74**	**3.69**	**1.47**	**143.58**	**39.92**	**2.54**	**5.13**	**30.37**	**320.2**
**Min (Overall)**	**0**	**0.4**	**0.01**	**0.02**	**0.04**	**0.12**	**0.04**	**10.6**	**5.77**	**0.65**	**0.56**	**6.07**	**71.15**
**Max (Overall)**	**9.66**	**15.75**	**0.69**	**210.45**	**7.38**	**7.73**	**2.74**	**228.76**	**92.68**	**4.96**	**37.55**	**76.22**	**485.01**

Each value represents the mean ± SD of three independent replications.

The results are presented as mg/kg rice flour, and values in each column with different capital letters are significantly different (P < 0.001) means of the three colored groups, while the different small letters in each column are significantly different (P < 0.001) content of phenolic acids in each colored group. GA, gallic acid: 2,5-DHA, 2,5-dihydroxybenzoic acid; VA, vanillic acid; p- HBA, p-hydroxybenzoic acid; SYA, syringic acid; CHA, Chlorogenic acid; t-CA, trans-cinnamic acid; t-FA, trans-ferulic acid; p-CA, p-coumaric acid; SIA, sinapic acid; KAF, kaempferol; CH, catechin hydrate. n.d: not detected.

Bold values represent the mean, range, and p-value of individual phenolic acids.

### Bran phenolic acids

GA existed in both the free and bound form in bran of black, red, and NP rice genotypes, with its content ranging from 2.12 to 4.58 and 6.4 to 9.66; 1.68 to 3.93 and 2.78 to 6.56; and 2.31 to 7.00 and 0.37 to 2.46 mg/kg, respectively (**Tables 4**, **5**) The highest content of GA (9.66mg/kg) was determined in the bound form in black rice genotype FCP. 2,5-DHA existed in both the free and bound form in bran of black, red, and NP rice genotype, with its content ranging from 0.11to 0.27 and 11.33 to 20.97; 0 to 0.23 and 5.03 to 23.9; and 0 to 0.24 and 1.21 to 8.26 mg/kg, respectively. The highest content (23.9mg/kg) of 2,5-DHA was determined in the bound form of red rice genotype DMI. VA existed in both the free and bound form in bran of black, red, and NP rice genotypes, with its content ranging from 0.16 to 1.29 and 0.06 to 0.7; 0.49 to 2.92 and 0.09to 11.42; and 0.16 to 4.21 and 0.08to 12.45 mg/kg, respectively. The highest content (12.45mg/kg) of VA was determined in the bound form of NP rice genotype PR121. *p*-HBA existed in both the free and bound form the in bran of black, red, and NP rice genotype, with its content ranging from 30.29 to 35.24 and 10.2 to 131.48; 6.58 to 44.23 and 8.58 to 206.9; and 7.54 to 44.23 and 2.17 to 232.22 mg/kg respectively. The highest content (232.22mg/kg) of *p*-HBA was determined in the bound form in NP rice genotype PB 1121. SYA existed in both the free and bound form in bran of black, red, and NP rice genotypes, with its content ranging from 246.54 to 266.73 and 0.24 to 3.15; 2.19 to 310.38 and 0.19 to 2.00; and 2.19 to 311.36 and 0.14to 1.60mg/kg, respectively. The highest content of SYA was determined in the free form in red rice genotype MSTY and NP rice genotype PR 126 (310.38 and 311.36mg/kg). Of the total 10 phenolic acids estimated, SYA was unlike the others, as it was found in higher volumes in the free form compared to the bound form. CHA existed in both the free and bound form in bran fraction of black, red, and NP rice genotypes, with its content ranging from 2.23 to 7.73 and 1.99 to 9.3; 1.88 to 9.15 and 3.36 to 8.32; and 0.52 to 9.16 and 0.57 to 4.25 mg/kg, respectively. The highest content of CHA was determined in the bound form in black rice genotype, CP (9.3mg/kg). The highest content of CHA was determined in the free form of NP rice, with the highest content of 9.16 mg/kg being present in PR 126. *trans*- CA existed in both the free and bound form in bran fraction of black, red, and NP rice genotypes, with its content ranging from 0.91 to 1.03 and 1.22 to 3.47; 0.01 to 1.11 and 0.45 to 2.56; and 0.01 to 3.89 and 0.19 to 3.47 mg/kg, respectively. The highest content of *trans*- CA was determined in the bound form of black rice genotype CP (3.47mg/kg) and in the free form of NP genotype Pb Bas 7(3.89mg/kg). *trans*- FA existed in both the free and bound form in the bran of black, red, and NP rice genotype, with its content ranging from 2.12 to 5.74 and 138.83 to 195.44; 1.75to 6.07 and 191.39 to 266.83; and 1.16 to 6.07 and 21.72 to 234 mg/kg, respectively. *trans*- FA was found to be high in all three color groups in the bound form of their bran fraction. The highest content of bound *trans*- FA was determined in black rice genotype FCAP (195.44 mg/kg), red rice genotype DMI (266.83 mg/kg), and NP rice genotype PR121 (234mg/kg). *p*-CA existed in both the free and bound form in bran of black, red, and NP rice genotypes, with its content ranging from 1.57 to 2.37 and 58.76 to 79.72; 1.06 to 2.19 and 58.2 to 88.08; and 0.52 to 2.19 and 4.04 to 57.47 mg/kg, respectively. The highest content of *p*-CA was determined in the bound form in the red rice genotype MSTY (88.08 mg/kg). SIA existed in both the free and bound form in bran fraction of black, red, and NP rice genotypes, with its content ranging from 1.28 to 1.91 and 2.34 to 6.81; 1.1 to 31.14 and 1.73 to 9.12; and 0.61 to 17.23 and 0.19 to 14.5 mg/kg, respectively. The highest content of SIA was determined in the free form in the red rice genotype YKM (31.14 mg/kg). KAF existed in both the free and bound form in bran of black, red, and NP rice genotypes, with its content ranging from 1.09 to 1.97 and 1.36 to 1.72; 1.31 to 75.52 and 1.36 to 1.83; and 0.93 to 105.81 and 0.76 to 33.6 mg/kg, respectively. The highest content of KAF was determined in the free form of NP rice genotype Bas 370 (105.81 mg/kg). Higher mean content of KAF was determined in the free form (23.42 mg/kg) compared to bound form (6.31 mg/kg). CH existed in both the free and bound form in bran of black, red, and NP rice genotypes with its content ranging from 3.29 to 11.09 and 33.65 to 87.36; 2.1to 11. and 30.13 to 70.33; and 1.92 to 11.60 and 14.45 to 39.07 mg/kg, respectively. The highest content of CH was determined in the bound form in the black rice genotype CP (87.36 mg/kg).

**Table 4:** The content of free phenolic acids in the bran.

Genotypes	Hydroxybenzoic acid				Hydroxycinnamic acid					Flavonoids			
Black rice	GA	2,5- DHA	VA	*p*-HBA	SYA	CHA	*t*-CA	*t*-FA	*p*-CA	SIA	KAF	CH	TOTAL
**FCP**	4.58a	0.23b	0.2b	30.29c	266.73a	7.73a	0.95b	5.32b	2.16b	1.91a	1.92a	11.09a	333.1b
**FCAP**	2.12c	0.11c	1.29a	35.24a	246.54b	2.23b	0.91b	2.12c	1.57c	1.62b	1.09b	3.29c	298.13c
**CP**	4.45b	0.27a	0.16b	32.94b	265.94a	7.73a	1.03a	5.74a	2.37a	1.28c	1.97a	10.38b	334.26a
**Mean**	**3.71^A^**	**0.20^A^**	**0.55^B^**	**32.82^A^**	**259.74^A^**	**5.90^A^**	**0.96^AB^**	**4.4^A^**	**2.03^A^**	**1.6^B^**	**1.66^B^**	**8.25^A^**	**321.83^A^**
**Range**	**2.12-4.58**	**0.11-0.27**	**0.16-1.29**	**30.29-35.24**	**246.54-266.73**	**2.23-7.73**	**0.91-1.03**	**2.12-5.74**	**1.57-2.37**	**1.28-1.91**	**1.09-1.97**	**3.29-11.09**	**298.13-334.26**
**P-Value**	**<0.001**	**<0.001**	**<0.001**	**<0.001**	**<0.001**	**<0.001**	**<0.001**	**<0.001**	**<0.001**	**<0.001**	**<0.001**	**<0.001**	**<0.001**
Red rice													
**GSL**	1.68e	n.d	2.92a	20.66e	179.05b	1.88e	0.25b	1.75e	1.52d	16.32b	3.41d	2.1d	231.54d
**DMI**	2.91b	0.19b	0.49f	6.58f	175.22c	3.06d	0.26b	3.32c	1.52d	16.31b	57.25b	4.89b	272c
**KMA**	1.87d	0.18c	0.82d	23.37d	103.01e	3.67c	0.16c	2.92d	2.01b	16.72b	46.75c	4.12c	205.6e
**BKG**	2.33c	0.1d	1.48b	44.23a	2.19f	2.91d	1.11a	2.8d	1.06e	1.48c	1.31d	4.00c	64.99f
**MSTY**	3.93a	0.03e	0.57e	34.57b	310.38a	9.15a	0.01d	6.07a	1.84c	1.1c	2.3d	11.57a	381.54a
**YKM**	2.35c	0.23a	0.97c	32.33c	149.01d	5.37b	0.24b	4.15b	2.19a	31.14a	72.52a	4.83b	305.33b
**Mean**	**2.51^B^**	**0.12^B^**	**1.21^A^**	**26.96^B^**	**153.14^B^**	**4.34^B^**	**0.34^B^**	**3.5^B^**	**1.69^AB^**	**13.85^A^**	**30.59^A^**	**5.25^B^**	**243.50^B^**
**Range**	**1.68-3.93**	**0-0.23**	**0.49-2.92**	**6.58-44.23**	**2.19-310.38**	**1.88-9.15**	**0.01-1.11**	**1.75-6.07**	**1.06-2.19**	**1.1-31.14**	**1.31-75.52**	**2.1-11.57**	**64.99-381.54**
**P-Value**	**<0.001**	**<0.001**	**<0.001**	**<0.001**	**<0.001**	**<0.001**	**<0.001**	**<0.001**	**<0.001**	**<0.001**	**<0.001**	**<0.001**	**<0.001**
Non-pigmented rice													
**PR 114**	4.55b	0.23b	0.2e	30.29c	267.2b	7.73b	0.95d	5.32b	1.65c	1.91d	1.92cd	11.09b	333.03b
**PR 121**	2.31e	0.1c	1.48c	44.23a	2.19g	2.91d	1.11c	2.8e	2.18a	1.48e	1.31de	4.00e	66.1g
**PR 126**	3.91c	0.05e	0.60d	33.49b	311.36a	9.16a	0.03f	6.09a	2.19a	1.3f	1.9c	11.60a	381.68a
**BAS 370**	3.07d	n.d	0.2ef	7.54f	3.32f	0.52f	2.64b	4.38d	1.03e	13.92c	105.81a	5.96d	148.39f
**BAS 386**	3.89c	0.24a	0.16f	27.71d	232.05c	6.8c	0.88d	4.77c	2.07b	0.61g	1.94cd	9.28c	290.4c
**PB 7**	7.00a	0.04d	4.21a	34.96b	6.65e	0.56f	3.89a	1.16f	1.22d	16.68b	72.08b	1.92f	150.39e
**PB 1121**	3.89c	n.d	1.68b	12.95e	176.48d	1.85e	0.2e	1.17f	0.52f	17.23a	0.93e	11.18b	228.06d
**Mean**	**4.09^A^**	**0.11^B^**	**1.24^A^**	**26.39^B^**	**143.59^B^**	**4.23^B^**	**1.4^A^**	**3.67^B^**	**1.55^B^**	**7.56^B^**	**26.21^AB^**	**7.89^AB^**	**228.32^C^**
**Range**	**2.31-7**	**0-0.24**	**0.16-4.21**	**7.54-44.23**	**2.19-311.36**	**0.52-9.16**	**0.01-3.89**	**1.16-6.07**	**0.52-2.19**	**0.61-17.23**	**0.93-105.81**	**1.92-11.60**	**66.1-381.86**
**P-Value**	**<0.001**	**<0.001**	**<0.001**	**<0.001**	**<0.001**	**<0.001**	**<0.001**	**<0.001**	**<0.001**	**<0.001**	**<0.001**	**<0.001**	**<0.001**
**Average (Overall)**	**3.43**	**0.12**	**1.09**	**28.28**	**168.52**	**4.58**	**0.91**	**3.74**	**1.69**	**8.8**	**23.42**	**6.95**	**251.54**
**Min (Overall)**	**1.68**	**0**	**0.16**	**6.58**	**2.19**	**0.52**	**0.01**	**1.16**	**0.52**	**0.61**	**0.93**	**1.92**	**64.99**
**Max (Overall)**	**7**	**0.27**	**4.21**	**44.23**	**310.38**	**9.15**	**3.89**	**6.07**	**2.37**	**31.14**	**105.81**	**11.57**	**381.86**

Each value represents the mean ± SD of three independent replications.

The results are presented as mg/kg rice flour, and values in each column with different capital letters are significantly different (P < 0.001) means of the three colored groups, while the different small letters in each column are significantly different (P < 0.001) content of phenolic acids in each colored group. GA, gallic acid: 2,5-DHA, 2,5-dihydroxybenzoic acid; VA, vanillic acid; p- HBA, p-hydroxybenzoic acid; SYA, syringic acid; CHA, Chlorogenic acid; t-CA, trans-cinnamic acid; t-FA, trans-ferulic acid; p-CA, p-coumaric acid; SIA, sinapic acid; KAF, kaempferol; CH, catechin hydrate. n.d: not detected.

Bold values represent the mean, range, and p-value of individual phenolic acids.

**Table 5:** The content of bound phenolic acids in the bran.

Genotypes	Hydroxybenzoic acid					Hydroxycinnamic acid					Flavonoids		
Black rice	GA	2,5-DHA	VA	*p*-HBA	SYA	CHA	*t*-CA	*t*-FA	*p*-CA	SIA	KAF	CH	TOTAL
**FCP**	9.66a	11.33c	0.7a	131.48a	1.5b	1.99c	1.22c	169.8b	79.72a	2.44b	1.44b	58.22b	469.49a
**FCAP**	7.5b	18.86b	0.06c	95.56b	3.15a	6.63b	2.24b	195.44a	58.76c	2.34b	1.36b	33.65c	425.55b
**CP**	6.4c	20.97a	0.48b	10.2c	0.24c	9.3a	3.47a	138.83c	71.33b	6.81a	1.72a	87.36a	357.1c
**Mean**	**7.85^A^**	**17.05^A^**	**0.42^B^**	**79.08^A^**	**1.63^A^**	**5.97^A^**	**2.31^A^**	**168.02^B^**	**69.94^A^**	**3.86^B^**	**1.51^B^**	**59.74^A^**	**417.38^B^**
**Range**	**6.4-9.66**	**11.33-20.97**	**0.06-0.7**	**10.2-131.48**	**0.24-3.15**	**1.99-9.3**	**1.22-3.47**	**138.83-195.44**	**58.76-79.72**	**2.34-6.81**	**1.36-1.72**	**33.65-87.36**	**357.1-469.49**
**P-Value**	**<0.001**	**<0.001**	**<0.001**	**<0.001**	**<0.001**	**<0.001**	**<0.001**	**<0.001**	**<0.001**	**<0.001**	**<0.001**	**<0.001**	**<0.001**
Red rice													
**GSL**	4.05c	5.03e	11.42a	21.91c	1.61b	3.75d	0.88d	221.7c	64.6d	9.12a	1.65b	32.56e	378.29e
**DMI**	3.58d	23.9a	0.32b	9.11d	1.6b	8.32a	2.56a	266.83a	80.47b	4.96b	1.82a	70.33a	473.8b
**KMA**	3.06e	5.96d	0.09c	206.9a	1.55bc	3.36e	0.45e	203.65d	63.25d	5.01b	1.45d	35.19d	529.91a
**BKG**	6.56a	19.26b	0.1c	52.99b	2a	7.47b	1.97c	191.39e	58.2e	2.05d	1.36e	30.13f	373.48e
**MSTY**	4.74b	18.2c	0.36b	8.58d	0.19d	6.44c	1.92c	242.39b	88.08a	3.01c	1.83a	54.49c	430.24c
**YKM**	2.78f	18.49c	0.31b	8.77d	1.5c	6.7c	2.21b	233.96b	67.86c	1.73e	1.54c	57.92b	403.78d
**Mean**	**4.13^B^**	**15.14^A^**	**2.10^AB^**	**51.38^C^**	**1.41^A^**	**6.00^A^**	**1.67^B^**	**226.65^A^**	**70.41^A^**	**4.31^AB^**	**1.61^B^**	**46.77^B^**	**431.58^A^**
**Range**	**2.78-6.56**	**5.03-23.9**	**0.09-11.42**	**8.58-206.9**	**0.19-2**	**3.36-8.32**	**0.45-2.56**	**191.39-266.83**	**58.2-88.08**	**1.73-9.12**	**1.36-1.83**	**30.13-70.33**	**373.48-529.91**
**P-Value**	**<0.001**	**<0.001**	**<0.001**	**<0.001**	**<0.001**	**<0.001**	**<0.001**	**<0.001**	**<0.001**	**<0.001**	**<0.001**	**<0.001**	**<0.001**
Non- pigmented rice													
**PR 114**	2.46a	8.26a	12.21b	5.73f	1.57a	3.62b	0.27e	213.31c	54.91b	1.32e	1.59d	37.19b	342.45c
**PR 121**	1.96b	7.44b	12.45a	21.93e	1.6a	3.3c	0.19f	234a	57.47a	1.23e	1.99d	38.01ab	381.57b
**PR 126**	n.d	1.58e	0.21e	2.17g	0.23d	2.35e	1.73a	21.72g	4.04g	2.36d	27.48b	15.63de	79.49g
**BAS 370**	0.56c	5.77d	0.21de	30.93d	1.48b	2.8d	0.95b	109.6d	43.62d	4.94c	0.76e	31.1c	232.73e
**BAS 386**	0.37d	1.21f	0.28cd	79.58c	1.01c	0.58f	0.95b	71.22f	10.15f	0.19f	19.6c	14.45e	199.58f
**PB 7**	0.53c	1.72e	0.35c	91.68b	0.14e	0.57f	0.65c	84.03e	13.63e	14.5a	33.6a	16.43d	257.83d
**PB 1121**	2.43a	6.62c	0.08f	232.22a	1.58a	4.25a	0.58d	225.66b	48.03c	11.79b	1.72d	39.07a	574.03a
**Mean**	**1.19^C^**	**4.66^B^**	**3.69^A^**	**66.32^B^**	**1.09^B^**	**2.49^B^**	**0.76^C^**	**137.08^B^**	**33.12^B^**	**5.19^A^**	**12.39^A^**	**27.41^C^**	**295.38^C^**
**Range**	**0-2.46**	**1.21-8.26**	**0.08-12.45**	**2.17-232.22**	**0.14-1.6**	**0.57-4.25**	**0.19-1.73**	**21.72-234**	**4.04-57.47**	**0.19-14.5**	**0.76-33.6**	**14.45-39.07**	**79.49-574.03**
**P-Value**	**<0.001**	**<0.001**	**<0.001**	**<0.001**	**<0.001**	**<0.001**	**<0.001**	**<0.001**	**<0.001**	**<0.001**	**<0.001**	**<0.001**	**<0.001**
**Average (Overall)**	**3.54**	**10.91**	**2.48**	**63.11**	**1.31**	**4.46**	**1.39**	**176.47**	**54.01**	**4.61**	**6.31**	**40.73**	**369.33**
**Min (Overall)**	**0**	**1.21**	**0.06**	**2.17**	**0.14**	**0.57**	**0.19**	**21.72**	**4.04**	**0.19**	**0.76**	**14.45**	**79.49**
**Max (Overall)**	**9.66**	**23.9**	**12.45**	**232.22**	**3.15**	**9.3**	**3.47**	**266.83**	**88.08**	**14.5**	**33.6**	**87.36**	**574.03**

Each value represents the mean ± SD of three independent replications.

The results are presented as mg/kg rice flour, and values in each column with different capital letters are significantly different (P < 0.001) means of the three colored groups, while the different small letters in each column are significantly different (P < 0.001) content of phenolic acids in each colored group. GA, gallic acid: 2,5-DHA, 2,5-dihydroxybenzoic acid; VA, vanillic acid; p- HBA, p-hydroxybenzoic acid; SYA, syringic acid; CHA, Chlorogenic acid; t-CA, trans-cinnamic acid; t-FA, trans-ferulic acid; p-CA, p-coumaric acid; SIA, sinapic acid; KAF, kaempferol; CH, catechin hydrate. n. d: not detected.

Bold values represent the mean, range, and p-value of individual phenolic acids.

### Free, bound, and total phenolic content, DPPH, and ABTS. + radical scavenging activity in bran

In the bran fraction of black rice genotypes, free phenolic content ranged from 296- 342 GAE mg/100g (**Table 6**). Among the black rice genotypes, FCAP displayed the highest content of free phenolics (342mg GAE/100g). In red rice genotypes, free phenolic content ranged from 188-369GAE mg/100g. MSTY possessed the highest content of free phenolics (369GAE mg/100g). In the NP rice genotypes, free phenolic content ranged from 167-268GAE mg/100g. PB 1121 had the highest free phenolic content of 268GAE mg/100g. Bound phenolic content in the bran fraction of black rice genotypes ranged from 2841-3467GAE mg/100g. Among the three black rice genotypes, FCAP displayed the highest content of 3467GAE mg/100g. In red rice genotypes, bound phenolic content ranged from 1698-3598GAE mg/100g. KMA possessed the highest content of 3598GAE mg/100 g bound phenolics. In the NP rice genotypes, bound phenolic content ranged from 301-674GAE mg/100g and PB 1121 had the highest content (674mg GAE/100g.) The TPC in black rice ranged from 3150-3809GAE mg/100g and the FCAP displayed the highest content of TPC (3809 GAE mg/100g). In red rice genotypes, TPC ranged from 1985-3851GAE mg/100g. KMA possessed the highest content of bound phenolics (3851 mg GAE/100g). In the NP rice genotypes, TPC ranged from 498-942GAE mg/100g. PB 1121 had the highest TPC of 942 GAE mg/100g.The contribution of bound phenolics to the TPC in all 16 rice genotypes under study was higher than free phenolics in both the bran and whole grain fraction (**Tables 6**, **7**) among the three colored-rice groups. In the black rice genotypes, the bran and whole grain bound phenolics contributed the maximum content of approximately 91%, followed by red rice (~89%) and NP genotypes toward TPC.

**Table 6:** The content of Free, Bound, and total phenolic content, DPPH, and ABTS.+ radical scavenging activity in bran.

Genotype	Phenolics (mg GAE/100 g)			DPPH (%of inhibition)		ABTS.+ (µM TE/g)	
	Free Phenolics	Bound Phenolics	TPC	Free Phenolics	Bound Phenolics	Free Phenolics	Bound Phenolics
**FCP**	309 ± 8.41b (1)	2841 ± 48.4c (90)	3150 ± 49.44c	43.7 ± 0.83b	80.9 ± 0.31a	21 ± 0.1b	24 ± 0.44a
**FCAP**	342 ± 12.02a (9)	3467 ± 96.88a (91)	3809 ± 86.43a	39.1 ± 0.37c	68.7 ± 0.72b	19 ± 0.57c	19 ± 0.41c
**CP**	296 ± 5.34b (9)	3045 ± 131.75b (91)	3341 ± 126.41b	49.8 ± 0.34a	82.7 ± 2.07a	26 ± 0.35a	21 ± 0.38b
**Mean**	**315.67^A^**	**3117.67^A^**	**3433.33^A^**	**44.2^A^**	**77.43^A^**	**22^A^**	**21.33^A^**
**Range**	**296-342**	**2841-3467**	**3150-3809**	**39.10-49.80**	**68.70-82.70**	**19-26**	**19-24**
**C.V.**	**2.86**	**3.16**	**2.71**	**2.19**	**2.85**	**1.77**	**1.93**
**P-Value**	**<0.001**	**<0.001**	**<0.001**	**<0.001**	**<0.001**	**<0.001**	**<0.001**
**GSL**	188 ± 7.46f (7)	2658 ± 62.29c (93)	2846 ± 58.92c	33.8 ± 0.32c	69.7 ± 1.27bc	1.4 ± 0.06e	11 ± 0.01d
**DMI**	213 ± 5.95e (9)	2198 ± 0d (91)	2411 ± 5.95d	29.7 ± 0.51d	71 ± 1.7b	1.36 ± 0e	15 ± 0.22b
**KMA**	253 ± 5.25d (7)	3598 ± 100.54a (93)	3851 ± 96.14a	35.4 ± 0.07b	58.2 ± 0.48d	8.1 ± 0.13b	14 ± 0.45c
**BKG**	348 ± 8.47b (15)	1902 ± 60.01e (85)	2250 ± 67.61e	29.8 ± 0.7d	66.4 ± 1.38c	2.5 ± 0.06d	16 ± 0.33a
**MSTY**	369 ± 3.99a (11)	2948 ± 7.97b (89)	3317 ± 4.82b	30.1 ± 0.06d	79.8 ± 1.5a	4 ± 0.15c	14 ± 0.03c
**YKM**	287 ± 9.31c (14)	1698 ± 29.08f (86)	1985 ± 30.87f	42.1 ± 0.35a	82.4 ± 1.72a	9 ± 0.06a	13.5 ± 0.47c
**Mean**	**276.33^B^**	**2500.33^B^**	**2776.67^B^**	**33.48^B^**	**71.25^B^**	**4.39^B^**	**13.92^B^**
**Range**	**188-369**	**1698-3598**	**1985-3851**	**29.70-42.10**	**58.20-82.40**	**1.36-9**	**11-16**
**C.V.**	**2.53**	**2.22**	**1.99**	**2.09**	**3.41**	**2.1**	**2.25**
**P-Value**	**<0.001**	**<0.001**	**<0.001**	**<0.001**	**<0.001**	**<0.001**	**<0.001**
**PR 114**	167 ± 6.32d (23)	560 ± 20.7bc (77)	727 ± 15.39c	50.7 ± 1.03a	75.8 ± 1.34a	0.8 ± 0.03c	14.8 ± 0.36b
**PR 121**	189 ± 2.9c (28)	479 ± 3.89d (72)	668 ± 3.5d	22.4 ± 0.31e	66.4 ± 0.86b	0.6 ± 0d	12.9 ± 0.23c
**PR 126**	169 ± 2.74d (23)	577 ± 13.52b (77)	746 ± 12.38b	30.8 ± 0.38d	69.2 ± 1.73b	0.9 ± 0.01b	19 ± 0.46a
**BAS 370**	200 ± 1.62b (34)	396 ± 0.36e (66)	596 ± 1.33e	44.1 ± 0.46b	58.7 ± 0.64c	0.91 ± 0.03b	10 ± 0.1f
**BAS 386**	169 ± 5.51d (24)	544 ± 6.86c (76)	713 ± 6.3c	21.4 ± 0.06e	68.2 ± 1.35b	1 ± 0.01a	11 ± 0.19e
**PB 7**	197 ± 3.91b (40)	301 ± 1.63f (60)	498 ± 4.17f	36.2 ± 0.53c	49.5 ± 0.41d	0.4 ± 0.02f	9 ± 0.03g
**PB 1121**	268 ± 2.66a (28)	674 ± 3.04a (72)	942 ± 3.96a	29.3 ± 0.72d	61.2 ± 1.31c	0.5 ± 0.02e	12 ± 0.21d
**Mean**	**194.14^C^**	**504.43^C^**	**698.57^C^**	**33.56^B^**	**64.14^C^**	**0.73^B^**	**12.67^B^**
**Range**	**167-268**	**301-674**	**498-942**	**21.40-50.70**	**49.50-75.80**	**0.4-1**	**9-19**
**C.V.**	**2.05**	**1.96**	**1.18**	**2.97**	**3.17**	**2.63**	**2.08**
**P-Value**	**<0.001**	**<0.001**	**<0.001**	**<0.001**	**<0.001**	**<0.001**	**<0.001**

Each value represents the mean ± SD of three independent replications.

The values in the parenthesis indicate the percent contribution of phenolics to the total phenolic content. The results are presented as GAE mg/100g, % inhibition, and µM TE/g rice bran, and values in each column with different capital letters are significantly different (P < 0.001). means of the three colored groups while the different small letters in each column are significantly different (P < 0.001) content of phenolic acids in each colored group. GAE, Gallic acid equivalent; µM TE, micromole Trolox equivalent.

Bold values represent the mean, range, and p-value of individual phenolic acids.

**Table 7:** The content of free, bound, and total phenolics, DPPH, and ABTS.+ radical scavenging activity in whole grain.

Samples	Whole grain Phenolics (GAE mg/100 g)			DPPH % of inhibition		ABTS.+ (µM TE/g)	
	Free Phenolics	Bound Phenolics	TPC	Free Phenolic	Bound Phenolic	Free Phenolics	Bound Phenolics
**FCP**	35 ± 0.92c (10)	326 ± 10.87c (90)	361 ± 11.79c	39.78 ± 0.37a	75.14 ± 1.58a	6 ± 0.13b	8 ± 0.12b
**FCAP**	37 ± 0.37b (8)	399 ± 7.19a (92)	436 ± 6.83a	35.14 ± 0.2b	65.92 ± 1.34b	3 ± 0.02c	6 ± 0.26c
**CP**	39 ± 0.91a (10)	348 ± 11.29b (90)	387 ± 12.21b	41.25 ± 0.73a	71.35 ± 0.89a	8 ± 0.13a	12 ± 0.44a
**Mean**	**37^A^**	**357.67^A^**	**394.67^A^**	**38.72^A^**	**70.8^A^**	**5.67^A^**	**8.67^A^**
**Range**	**35-39**	**326-399**	**361-436**	**35.14-41.25**	**65.92-75.14**	**3-8**	**6-12**
**C.V.**	**2.1**	**2.78**	**2.68**	**2.18**	**3.18**	**1.88**	**3.52**
**P-Value**	**<0.001**	**<0.001**	**<0.001**	**<0.001**	**<0.001**	**<0.001**	**<0.001**
**GSL**	18 ± 0.5f (6)	298 ± 12.09b (94)	316 ± 12.59c	30.2 ± 0.41c	63.14 ± 0.13d	0.16 ± 0f	2.4 ± 0.05d
**DMI**	22 ± 0.61e (8)	255 ± 4.6c (92)	277 ± 5.13d	25.6 ± 0.27e	75.14 ± 1.29b	0.19 ± 0e	3 ± 0.13ab
**KMA**	30 ± 0.49c (7)	409 ± 10.32a (93)	439 ± 10.09a	32.46 ± 0.56b	61.29 ± 0.35d	1.2 ± 0.03b	2.9 ± 0.02bc
**BKG**	48 ± 0.95a (19)	199 ± 2.69d (81)	247 ± 3.56e	28.7 ± 0.39d	67.16 ± 1.19c	0.5 ± 0.02c	3.1 ± 0.03a
**MSTY**	44 ± 0.83b (12)	310 ± 9.5b (88)	354 ± 8.81b	30.5 ± 0.44c	75.12 ± 0.39b	0.4 ± 0d	2.8 ± 0.01c
**YKM**	27 ± 0.17d (13)	188 ± 6.44d (87)	215 ± 6.44f	37.6 ± 0.08a	79.54 ± 1.28a	1.3 ± 0.02a	2.5 ± 0.01d
**Mean**	**31.5^A^**	**276.5^B^**	**308^B^**	**30.84^B^**	**70.23^A^**	**0.62^B^**	**2.78^B^**
**Range**	**18-48**	**188-409**	**215-439**	**25.60-37.60**	**61.29-79.54**	**0.16-1.3**	**2.4-3.1**
**C.V.**	**2.05**	**3**	**2.71**	**2.18**	**2.26**	**2.5**	**2.15**
**P-Value**	**<0.001**	**<0.001**	**<0.001**	**<0.001**	**<0.001**	**<0.001**	**<0.001**
**PR 114**	22 ± 0.58d (25)	66 ± 0.12b (75)	88 ± 0.53b	48.21 ± 0.38a	70.98 ± 1.22a	0.12 ± 0c	1.7 ± 0.01d
**PR 121**	16 ± 0.12g (24)	51 ± 0.37d (76)	67 ± 0.38d	19.4 ± 0.29f	61.21 ± 0.32cd	0.06 ± 0f	1.1 ± 0.03f
**PR 126**	19 ± 0.58e (22)	67 ± 0.36b (78)	86 ± 0.83b	25.9 ± 0.62e	59.75 ± 0.19d	0.13 ± 0b	2.2 ± 0a
**BAS 370**	24 ± 0.17b (35)	44 ± 0.24e (65)	68 ± 0.41d	39.7 ± 0.21b	63.41 ± 1.06c	0.11 ± 0d	1.9 ± 0.05b
**BAS 386**	18 ± 0.41f (23)	60 ± 1.19c (77)	78 ± 1.6c	20.1 ± 0.08f	67.41 ± 1.05b	0.14 ± 0a	1.1 ± 0.04f
**PB 7**	23 ± 0.81c (38)	38 ± 0.75f (62)	61 ± 1.52e	32.91 ± 0.82c	38.11 ± 0.87f	0.06 ± 0f	1.5 ± 0.05e
**PB 1121**	29 ± 0.03a (24)	91 ± 3.2a (76)	120 ± 3.22a	27.8 ± 0.16d	55.8 ± 0.99e	0.07 ± 0e	1.8 ± 0.03c
**Mean**	**21.57^B^**	**59.57^C^**	**81.14^C^**	**30.57^B^**	**59.52^B^**	**0.1^B^**	**1.61^B^**
**Range**	**16-29**	**38-91**	**61-120**	**19.40-48.21**	**38.11-70.98**	**0.06-0.14**	**1.1-2.2**
**C.V.**	**2.17**	**2.25**	**1.89**	**2.50**	**2.60**	**3.05**	**2.24**
**P-Value**	**<0.001**	**<0.001**	**<0.001**	**<0.001**	**<0.001**	**<0.001**	**<0.001**

Each value represents the mean ± SD of three independent replications.

The values in the parenthesis indicate the percent contribution of the free and bound phenolic fraction to the total phenolic content The results are presented as GAE mg/100g, % inhibition and µM TE/g whole grain, and values in each column with different capital letters are significantly different (P < 0.001). means of the three colored groups while the different small letters in each column are significantly different (P < 0.001) content of phenolics and antioxidant activity in each colored group. GAE, Gallic acid equivalent; µM TE, micromole Trolox equivalent.

Bold values represent the mean, range, and p-value of individual phenolic acids.

In the bran fraction of black rice genotypes, DPPH activity of free phenolics ranged from 39.10-49.80%. Among the black rice genotypes, CP displayed the highest DPPH activity of 49.80%. In red rice genotypes, DPPH activity in free phenolics ranged from 29.70-42.10%. YKM possessed the highest DPPH activity (42.10%.) In the NP rice genotypes, DPPH activity of free phenolics ranged from 21.40-50.70%. PR 114 had the highest DPPH activity of 50.70%. In the bran fraction of black rice genotypes, DPPH activity of bound phenolics ranged from 68.70-82.70%. Among the black rice genotypes, CP displayed the highest DPPH activity of 82.70%. In red rice genotypes, DPPH activity of bound phenolics ranged from 58.20- 82.40%. YKM possessed the highest DPPH activity (82.40%.) In the NP rice genotypes, DPPH activity of bound phenolics ranged from 49.50-75.80%. PR 114 had the highest DPPH activity of 75.80%. Significantly, for the DPPH activity of bran phenolics of NP rice genotypes, PR114 was comparable to the mean of DPPH activity in black rice genotypes (**Table 6**). In the bran fraction black rice genotypes, ABTS. + activity in free phenolics ranged from 19-26(µM TE/g) Among the three black rice genotypes, CP displayed the highest ABTS. + activity of 26 µM TE/g). In red rice genotypes, ABTS. + activity ranged from 1.36-9µM TE/g. YKM possessed the highest ABTS. + activity of 9µM TE/g. In the NP rice genotypes, ABTS. + activity ranged from 0.4-1.0µM TE/g. BAS 386 had the highest ABTS. + activity of 1µM TE/g. In the bran fraction of black rice genotypes, ABTS. + activity of bound phenolics ranged from 19-24µM TE/g. Among the black rice genotypes, FCP displayed the highest ABTS. + activity of 24 µM TE/g. In red rice genotypes, ABTS. + activity ranged from 11-16µM TE/g. BKG possessed the highest ABTS. + activity of 16 µM TE/g. In the NP rice genotypes, ABTS. + activity ranged from 9-19µM TE/g. PR 126 had the highest ABTS. + activity of 19 µM TE/g. Notably ABTS.+ activity of the bran-bound phenolics of NP rice genotypes PR 126 was significantly higher than the mean ABTS.+ activity of red rice genotype.

### Free, bound, and total phenolics, DPPH, and ABTS. + radical scavenging activity in whole grain

In the whole grain fraction of black rice genotypes, free phenolic content ranged from 35-39GAEmg/100g. (**Table 7**), with the black rice genotype CP having the highest content of 39 GAE mg/100g. In red rice genotypes, free phenolic content ranged from 18-48GAE mg/100g. Red rice genotype BKG possessed the highest content of 48GAE mg/100g. In NP rice genotypes, the content of free phenolic ranged from 16-29GAE mg/100g. PB 1121 possessed the highest free phenolic content of 29 GAE mg/100g. Bound phenolic content of whole grain black genotypes ranged from 326-399GAE mg/100g, with the black rice genotype FCAP having the highest content (399 GAE mg/100g). In the red rice genotypes, bound phenolic content ranged from 188-409GAE mg/100g. Red rice genotype KMA possessed the highest content of 409 GAE mg/100g. In NP rice genotypes, the content of bound phenolics ranged from 38-91GAE mg/100g. PB 1121 possessed the highest bound phenolic content of 91GAE mg/100g. TPC of whole grain fraction of black rice genotypes ranged from 361-436GAE mg/100g. Among the black rice genotypes, FCAP displayed the highest content of 436GAE mg/100g. In the red rice genotypes, TPC ranged from 215-439GAE mg/100g. KMA possessed the highest content of 439GAE mg/100g. In the NP rice genotypes, TPC ranged from 61-120 GAE mg/100g. PB 1121 had the highest TPC at 120 GAE mg/100g. Among the three colored-rice groups, black rice possessed significantly higher whole grain TPC than red and NP rice genotypes.

In the whole grain fraction of black rice genotypes, DPPH activity of free phenolics ranged from 35.14-41.25%. Among the three black rice genotypes, CP displayed the highest DPPH activity of 41.25%. In red rice genotypes, DPPH activity of free phenolics ranged from 25.60- 37.60%. YKM possessed the highest DPPH activity of 37.60%. In the NP rice genotypes, DPPH activity of free phenolics ranged from 19.40-48.21%. PR 114 had the highest DPPH activity of 48.21%. In the whole grain fraction of black rice genotypes, DPPH activity of bound phenolics ranged from 65.92-75.14%. Among the three black rice genotypes, FCP displayed the highest DPPH activity of 75.14%. In red rice genotypes, DPPH activity ranged from 61.29-79.54%. YKM possessed the highest DPPH activity at 79.54%. In the NP rice genotypes, DPPH activity of bound phenolics ranged from 38.11-70.98%. PR 114 had the highest DPPH activity of 70.98%. In whole grain fraction of black rice genotypes, ABTS. + activity of free phenolics ranged from 3-8µM TE/g. Among the black rice genotypes, CP displayed the highest ABTS. + activity of 8 µM TE/g. In red rice genotypes, ABTS. + activity ranged from 0.16-1.3 µM TE/g. YKM possessed the highest ABTS. + activity (1.3µM TE/g). In the NP rice genotypes, ABTS. + activity ranged from 0.06-0.14µM TE/g. BAS 386 had the highest ABTS. + activity of 0.14 µM TE/g. In the whole grain fraction of black rice genotypes, ABTS. + activity of bound phenolics ranged from 6-12µM TE/g. Among the black rice genotypes, CP displayed the highest ABTS. + activity (12 mg µM TE/g). In red rice genotypes, ABTS. + activity ranged from 2.4-3.1µM TE/g. BKG possessed the highest ABTS.+ activity of 3.1 µM TE/g. In NP rice genotypes, ABTS.+ activity ranged from 1.1-2.2µM TE/g. PR 126 had the highest ABTS.+ activity of 2.2 µM TE/g.

### Anthocyanins, proanthocyanidins, and flavonoids content in bran and whole grain

Anthocyanins are natural plant pigments. Glycosides are composed of the anthocyanidin aglycone with one or more glycosidically bonded mono or oligosaccharide units that have beneficial effects for the plants as well as for humans and animals. In bran fraction black rice genotypes, anthocyanin content ranged from 1102-2364 mg GAE/100g (**Table 8**). Among the three black rice genotypes, FCAP displayed the highest content of anthocyanins (2364 mg GAE/100g, **Table 8**) In red rice genotypes, anthocyanin content ranged from 22-33 mg GAE/100g. Out of six red rice genotypes, KMA possessed the highest content of 33 mg GAE/100g anthocyanins. In the NP rice genotypes, anthocyanin content ranged from 3.1-22 mg GAE/100g. PR 126 had the highest anthocyanin content of 22 mg GAE/100g. In the whole grain fraction of black rice genotypes, anthocyanin content ranged from 189-239 mg GAE/100g. And the black genotype CP displayed the highest content of anthocyanin (239 mg GAE/100g). In red rice genotypes, anthocyanin content ranged from 2.5-.3.9 mg GAE/100g. Red rice genotype KMA displayed the highest content of 3.9 mg GAE/100g. In NP rice genotypes, anthocyanin content ranged from 0.4-2.98 mg GAE/100g and PR126 displayed the highest content of 2.98 mg GAE/100g.

**Table 8:** The content of anthocyanins, proanthocyanidins, and flavonoids in bran and whole grain.

Genotypes	Anthocyanin (C3G mg/100g)		Proanthocyanidins (CAE mg/100g)		Flavonoids (QE mg/100g)	
Black rice	Bran	Whole grain	Bran	Whole grain	Bran	Whole grain
**FCP**	1102 ± 20.86c	189 ± 0.34c	76 ± 2.04c	11 ± 0.12c	1236 ± 41.22	384 ± 14.88a
**FCAP**	2364 ± 27.7a	220 ± 2.58b	146 ± 3.68a	14 ± 0.1a	1187 ± 21.4	359 ± 12.94b
**CP**	2171 ± 13.7b	239 ± 7.97a	98 ± 3.89b	13 ± 0.4b	1289 ± 53.45	405 ± 3.65a
**Mean**	**1879^A^**	**216^A^**	**106.67^B^**	**12.67^B^**	**1237.33^A^**	**382.67^A^**
**Range**	**1102-2364**	**189-239**	**76-146**	**11-14**	**1187-1289**	**359-405**
**C.V.**	**1.15**	**2.24**	**3.1**	**1.95**	**3.3**	**3.03**
**P-Value**	**<0.001**	**<0.001**	**<0.001**	**<0.001**	**NS**	**<0.001**
Red rice						
**GSL**	29 ± 0.16c	3.1 ± 0.12c	908 ± 14.73e	86 ± 1.71e	905 ± 25.94d	399 ± 7.41a
**DMI**	31 ± 0.45b	2.9 ± 0.04d	2431 ± 52.59a	204 ± 8.46a	1022 ± 20.27b	350 ± 13.25b
**KMA**	33 ± 1.07a	3.9 ± 0.04a	1063 ± 45.99d	99 ± 0.89d	865 ± 5.46e	258 ± 3.95d
**BKG**	26 ± 0.14e	3.1 ± 0.1c	1874 ± 62.5c	169 ± 5.79c	1109 ± 30.99a	298 ± 12.09c
**MSTY**	22 ± 0.24f	2.5 ± 0.11e	694 ± 11.89f	77 ± 0.14e	745 ± 2.01f	302 ± 6.81c
**YKM**	27 ± 0.24d	3.6 ± 0.12b	2048 ± 29.54b	182 ± 7.05b	970 ± 10.49c	288 ± 4.67c
**Mean**	**28^B^**	**3.18^B^**	**1503^A^**	**136.17^A^**	**936^B^**	**315.83^B^**
**Range**	**22-33**	**2.5-.3.9**	**694-2431**	**77-204**	**745-1109**	**258-399**
**C.V.**	**1.79**	**2.98**	**2.72**	**3.78**	**2.04**	**2.77**
**P-Value**	**<0.001**	**<0.001**	**<0.001**	**<0.001**	**<0.001**	**<0.001**
Non- pigmented rice						
**PR 114**	3.1 ± 0.06g	0.4 ± 0.003d	1.9 ± 0.06e	0.3 ± 0c	689 ± 28.57de	121 ± 3.71b
**PR 121**	8 ± 0.35e	0.9 ± 0.05cd	2.1 ± 0.05d	0.24 ± 0.01d	711 ± 3.2d	109 ± 3.64c
**PR 126**	22 ± 0.69a	2.98 ± 0.04a	3.4 ± 0.03ab	0.44 ± 0.01a	677 ± 9.15e	112 ± 3.43c
**BAS 370**	18 ± 0.41b	2.01 ± 0.02ab	2 ± 0.02de	0.1 ± 0f	936 ± 5.91b	99 ± 1.61d
**BAS 386**	14 ± 0.3c	1.56 ± 0.01b	2.8 ± 0.1c	0.14 ± 0e	1001 ± 23.46a	133 ± 5.63a
**PB 7**	9 ± 0.04d	1.14 ± 0.001c	3.5 ± 0.11a	0.4 ± 0b	889 ± 4.81c	89 ± 3.53e
**PB 1121**	3.9 ± 0.06f	0.51 ± 0.007d	3.3 ± 0.1b	0.1 ± 0f	1028 ± 26.87a	90 ± 1.7e
**Mean**	**11.14^B^**	**1.36^B^**	**2.71^C^**	**0.25^C^**	**847.29^B^**	**107.57^C^**
**Range**	**3.1-22**	**0.4-2.98**	**1.9-3.5**	**0.1-0.44**	**677-1028**	**89-133**
**C.V.**	**3.16**	**2.89**	**2.78**	**2.1**	**2.11**	**3.3**
**P-Value**	**<0.001**	**<0.001**	**<0.001**	**<0.001**	**<0.001**	**<0.001**

Each value represents the mean ± SD of three independent replications.

The results are presented as C3G mg/100g, CAE mg/100g and QE mg/100g rice bran and wholegrain values in each column with different capital letters are significantly different (P < 0.001). means of the three colored groups while the different small letters in each column are significantly different (P < 0.001) content of phenolic acids in each colored group. C3G, cyanidin-3-glucoside equivalents; CAE, Catechin equivalents; QE, quercetin equivalents.

Bold values represent the mean, range, and p-value of individual phenolic acids.

Proanthocyanidins, known as condensed tannins, are oligomers or polymers of flavan-3- ol units. These have a chemopreventive capacity and reduce the risk of cardiovascular mortality. In the bran fraction of black rice genotypes, the content of proanthocyanidins ranged from 76-146 mg CAE/100g. Among the three black rice genotypes, FCAP displayed the highest content of 2364 mg CAE/100g of proanthocyanidins. In the red rice genotypes, proanthocyanidin content ranged from 694-2431 mg CAE/100g. Red rice genotype DMI possessed the highest content of 2431 mg CAE/100g. In NP rice genotypes, the content of proanthocyanidins ranged from 1.9-3.5mg/100g. Pb Bas 7 displayed the highest proanthocyanidin content of 3.5 mg CAE/100g. In whole grain fraction of black rice genotypes, proanthocyanidin content ranged from 11-14 mg CAE/100g. The black rice genotype, FCAP, displayed the highest content of 14 mg CAE/100g. In red rice genotypes, proanthocyanidin content ranged from 77-204mg CAE/100g. Red rice genotype DMI possessed the highest content of 204 mg CAE/100g. In NP rice genotypes, proanthocyanidin content ranged from 0.1-0.44 mg CAE/100g. PR126 displayed the highest proanthocyanidin content of 0.44 mg CAE/100g. Flavonoids are secondary metabolites found in the form of aglycones or glycosides in many fruits and vegetables, and are believed to provide health benefits through a variety of signaling pathways and antioxidant effects. In the bran fraction black rice genotypes, flavonoid content ranged from 1187-1289 mg/100g. Among the three black rice genotypes, CP displayed the highest content of 1289 mg/100g. In red rice genotypes, flavonoid content ranged from 745-1109 mg/100g. Red rice genotype DMI possessed the highest content of 1109 mg/100g. In NP rice genotypes, flavonoid content ranged from 677-1028mg/100g, with PB 1121 displaying the highest content of 1028 mg/100g. In the whole grain fraction of black rice genotypes, flavonoid content ranged from 359-405mg/100g, with the black rice genotype CP having the highest content of 405 mg/100g. In red rice genotypes, flavonoid content ranged from 359-405mg/100g. Red rice genotype GSL possessed the highest content of 405 mg/100g. In NP rice genotypes, the content of flavonoids ranged from 258-399mg/100g. BAS 386 possessed the highest flavonoid content of 399 mg/100g.

Very fewer studies have investigated the correlation between individual phenolic acid, TPC, and their antioxidant activity. In this study, we attempted to fill this knowledge gap in regards to pigmented rice (**Supplementary Tables 1**, **2**). In black rice genotypes, whole grain- free TPC had a significant positive correlation with VA, while SIA had a negative correlation (p ≤ 0.01), and DPPH had a positive correlation with GA, VA, SYA, p-CA, and CH. P-HBA and SYA had a positive correlation with ABTS.+, while SIA had a negative correlation. In the bran fraction of NP rice, 2,5-DHA had a significant positive correlation with bound TPC and DPPH activity. GA, 2,5-DHA, trans-CA, and trans-FA contributed a negative correlation with bound TPC and DPPH antioxidant activity.

## Discussion

Phenolic acids and their derivatives are secondary metabolites widely distributed in fruits, vegetables, and cereal grains. In rice, phenolics exist in free and bound forms. Free phenolics are present within the plant cell vacuoles, while the bound form is present in the cell walls (Sumczynski et al., 2016).

Nearly 70–90% of the total phenolic acids found in whole grains are found in rice bran, where phenolic acids are concentrated at the grain’s periphery (Zhou et al., 2004). In the present study, most phenolic acids were found in the bound (insoluble) form (**Tables 2**–**5**). GA existed in both free and bound forms in bran and whole grain fraction of pigmented rice. However, it was not detected in the whole grain of NP genotype Bas 386, whereas very low content was detected in other NP genotypes under study (**Table 3**). The black rice genotype FCP displayed the highest content of GA in bound form in the bran fraction (**Table 5**). Shao et al. (2014) and Pang et al. (2018) have also reported that black rice had the highest GA content in bran compared to red and white rice. The free form of 2,5- DHA was not found in the bran fraction of NP genotypes Bas 370, Bas 386, and PB 1121, or in the red rice genotype GSL (**Table 4**). The average content of bound form 2,5-DHA in black rice bran fraction was higher (17.05mg/kg) than in red rice genotypes (15.14 mg/kg), with the exception of red rice genotype DMI, which displayed higher content (23.9 mg/kg) (**Table 5**) It is noteworthy that 2,5-DHA in rice grains has been reported in very few studies (Pang et al., 2018). Our findings are close to those reported by Pang et al. (2018), who found that the average content of 2,5-DHA was higher in black rice compared to red rice, with an exception of the red rice variety BX 105 which had higher content than black rice. VA existed in free and bound form in whole grain and bran fraction in pigmented and non-pigmented rice. The NP genotype PR 121 displayed the highest content (12.45mg/kg) of bound VA in bran fraction, followed by the red rice genotype GSL (11.42mg/kg). Miyazawa et al. (2003) and Kaur et al. (2018) stated that there are limited reports on the occurrence of VA in bound fractions. We found a low content of free VA in the whole grain of NP but Shao et al. (2014) did not detect a free form of VA in non-pigmented rice. Shao et al. (2014) stated that VA was found only in black rice, where it peaked at maturity. VA could be quantified only in black rice (Das et al., 2016). A low content of VA could be detected in both pigment and NP genotypes under study (**Tables 2**–**5**). The biotic stress conditions, such as infection by pathogens and parasites or wounding, and abiotic stress conditions such as exposure to extreme temperatures, air pollution, and UV radiation, might be responsible for the discrepancy in our findings with other research. The level of phenolics in grains also depends on factors such as cultivation and breeding techniques, growing conditions (e.g., altitude and fertilization), ripening process, and processing and storage conditions (Naczk and Shahidi, 2006; Min et al., 2012). It is generally known that different growing conditions and climates can influence phenolic content and composition as well as antioxidant activity (Sumczynski et al., 2016). A significant amount of *p*-HBA was determined in both free and bound form in bran and whole grain fraction of pigmented and NP rice genotypes under study. The whole grain black rice genotype FCP contained the highest amount of bound p-HBA (210.46 mg/kg). Bran of red rice genotype KMA and NP genotype PB 1121 possessed the highest amount of bound *p*-HBA (206.9 and 232.22 mg/kg respectively). A significant amount of *p*-HBA in bran, with an overall mean content of 28.28 and 63.11 mg/kg in free and bound form respectively, was obtained in the present study (**Tables 4**, **5**). Chaiyasut et al. (2015) did not find a detectable level of *p*-HBA while Huang and Lai (2016) detected it as a minor compound in free and bound form (7µg/g and 34µg/g respectively), while the purple and black rice bran extract were dominated by *p*-HBA (349 and 347g/L). The varying results might be due to varietal differences and different extraction procedures that were followed. The whole grain of NP genotype PR126 displayed the highest content, (182.39 mg/kg) of the free form of SYA among NP genotypes. The SYA amount quantified in the previous studies was quite low, ranging from 17.36 to 45.89 µg/g in wild rice (Qiu et al., 2010) to 2.65 to 4.74 µg/g in whole grain in bound form (Gong et al., 2017). also stated that the bound form of SYA in *japonica* was significantly higher than that in *Indica* rice. The NP genotype PR126, red rice genotype MSTY, and black rice genotype FCP displayed a high amount of free form SYA in the bran (311.36, 310.38, and 266.73 mg/kg respectively, **Table 4**). CHA was reported to exist only in free form by Ti et al. (2014) and Huang and Ng (2012), whereas we detected CHA in both free and bound forms in both bran and whole grain fraction (**Tables 2**–**5**). The black rice genotype FCAP possessed the highest amount of free CHA (7.71 mg/kg), while the red rice genotype MSTY displayed the highest amount of bound CHA (7.85 mg/kg) in the whole grain fraction. The black rice genotype CP displayed the highest amount of bound CHA in the bran fraction, 9.3mg/kg (**Table 6**), and NP genotype PR 126 displayed the highest amount of CHA in free form in the bran fraction. (9.16mg/kg). A high level of *t-*FA was detected in bound form in both whole grain and bran (**Tables 3**, **5**). The whole grain of black rice FCP and red rice MSTY displayed the highest content of *t-*FA in bound form (239.35 and 230.34 mg/kg). Similar reports by Shao et al. (2014) stated that pigmented rice possessed higher ferulic acid than NP rice. It has been reported that ferulic acid was the predominant phenolic acid in rice (Shao et al., 2014; Zaupa et al., 2015; Zhang et al., 2015). This observation was corroborated in the present study. *p*-CA was present in the free and bound form in both pigmented and NP rice. The whole grain of black rice genotype FCAP and red rice MSTY displayed the highest content of bound *p*-CA (49.65 and 98.44 mg/kg). The black genotype FCP and red rice genotype MSTY displayed the highest content of *p*-CA in bound form (79.72 and 88.08 mg/kg) in the bran fraction. (Ti et al., 2014) reported that CA existed in both free and bound form, its content ranging from 2.1 to 3.8 and from 6.0 to 14.7 µg/g respectively, and the average percentage contribution of bound CA to the total was 76.5%. Pigmented rice landraces were reported to possess higher amounts of bound *p*-CA (Kaur et al., 2018). These observations were also corroborated in the present study. Whole grain NP PR 114 displayed the highest content of free SIA (127.72 mg/kg), while the red rice genotype YKM displayed the highest content of free SIA in bran fraction. (31.14mg/kg) (Gong et al., 2017; Park et al., 2012) reported that SIA was detected in quite low amounts (1.19 and 1.25 µg/g, respectively) in whole grain. The content detected in this study was, however, higher than the quantity reported in these studies. This discrepancy may be due to differences in the genotype’s environmental conditions, agronomic practices, or extraction methods (Naczk and Shahidi, 2006; Shao and Bao, 2015; Pang et al., 2018).

Two flavonoids, KAF and CH, were detected. KAF was mainly found in free form and CH was predominant in bound form. (**Tables 2**–**5**) The whole grain of red rice MSTY and bran of black rice CP displayed the highest content of bound CH 82.29 and 87.36mg/kg, respectively (Huang and Ng, 2012; Praveen et al., 2016). stated that free-form CH is significant when compared to bound form in bran fraction. Praveen et al. (2016) employed a rapid non-aqueous capillary electrophoretic (NACE) system to quantify catechin (5.63 ± 1.52 μg g−1) and KAF (4.95 ± 2. 0 μg g−1), whereas Das et al. (2016) employed an ultrasound-assisted extraction (UAE) (±) technique for the extraction of CH from black and purple rice bran. The content of CH was 141.7 and 5.7 μg/L, respectively. The discrepancy in the results might be due to different methods employed for the extraction and estimation procedures.

### Total phenolic content and antioxidant activity

It can be seen from **Tables 6**, **7** that the contribution of bound phenolics to the TPC was much higher than free phenolics in both bran and whole grain. The bound TPC content in the bran ranged between 2841-3467 mg GAE/100g in black rice, 1698-3598mg GAE/100g in red rice, and 301-674 GAE/100g in NP rice. Meanwhile, in the whole grain fraction, the bound TPC content ranged between 326-399 mg GAE/100g in black rice from 188-409mg GAE/100g in red rice and 38-91GAE/kg in NP rice. The free form TPC content in bran ranged between 296-342 mg GAE/100g in black rice, from 188-369 mg GAE/100g in red rice, and 167-268 GAE/100g in rice bran. The free TPC content in whole grain ranged between 35-39 mg GAE/100g in black rice, 18-48mg GAE/100g in red rice, and 16-29 GAE/100g in NP rice. The highest TPC was recorded in bran fraction in black rice genotypes, specifically in FCAP followed by CP, at 3809 and 3341 mg GAE/100g, respectively, while in the red rice genotype the highest was found in KMA followed by MSTY, at 3851 and 3317 GAE mg/100g respectively. With regard to NP genotype, PB 1121 followed by PR 126 displayed the highest content at 942 and 746 GAE/100g respectively. The TPC values measured in rice bran were significantly higher than in whole grain rice. This is expected, since it has been reported that the phenolic constituents are predominantly distributed in the external layers of the rice grain Verardo et al. (2016). Liyana-Pathirana and Shahidi (2006) reported that bound phenolics’ contribution to the total phenolic content was significantly higher than that of free and esterified fractions in hard and soft whole wheat. Yao et al. (2010), found that the TPC of black rice was greater than that of red rice; similar results were found in our study. Apart from rice crop, other cereal crops also show that the major portion of phenolics in grains existed in the bound form (75% in oats and wheat, 85% in corn, and 62% in rice) Adom and Liu (2002). On the contrary, there are several reports stating that a higher amount of free phenolics than bound phenolics were present (Min et al., 2012; Sumczynski et al., 2016). Zhang et al. (2015), also found that free phenolics content was higher than the bound phenolics content in most of the breeding lines derived from a cross between white and black rice. The discrepancy in the results might be due to the distribution of phenolics in grains at the subcellular and cellular level, which is variable. It is well documented that different growing conditions and climates can influence phenolic contents and compositions as well as antioxidant activity (Sumczynski et al., 2016).

The determination of antioxidant activity by the DPPH radical scavenging method is considered a good *in-vitro* model to check the efficiency of the sample within a short period of time. The DPPH method is based on the mechanism of electron transfer and determines the reducing capacity of antioxidants. The DPPH antioxidant activity of the bound form of phenolics in bran ranged from 68.70-82.70%,58.20-82.40%, and 49.50-75.80% in black, red, and NP rice bran, respectively (**Table 7**) Among the black rice genotypes, FCP and CP displayed high DPPH activity in the bound form of phenolics (80.9% and 82.7% respectively). Red rice genotypes YKM, MSTY, and DMI displayed high DPPH activity of 82.4%,79.8%, and 71.0%, respectively. The higher DPPH activity in black and red-colored rice cultivars could be attributed to the higher anthocyanin and proanthocyanidin content, respectively. NP rice genotypes PR 114 and PR 126 possessed the highest DPPH activity of 75.8% and 69.2%, respectively, in the bound form of total phenolics from the bran. In whole grain, the DPPH activity in bound phenolics ranged from 65.92 -75.14%, 61.29-79.54, and 38.11-70.98% in black, red, and NP rice respectively. Among the black rice genotypes, FCP and CP displayed the highest DPPH activity of 75.14% and 71.35% respectively in bound phenolics in whole grain fraction. Red rice genotype YKM displayed the highest DPPH activity of 79.54% in bound phenolic fraction of whole grain. NP rice genotype PR 114 had the highest DPPH activity of 70.98%. It has been reported that the bound phenolic fraction demonstrated a significantly higher antioxidant capacity than free phenolics in hard and soft whole wheat (Liyana-Pathirana and Shahidi, 2006). In the case of free form, the DPPH activity was 2-3-fold less when compared to bound form in both bran and whole grain. Adom and Liu (2002), stated that the bound phytochemicals were the major contributors to the total antioxidant activity in cereals: 90% in wheat, 87% in corn, 71% in rice, and 58% in oats. Our study supported the conclusion of (Liyana-Pathirana and Shahidi, 2006; Pang et al., 2018), who suggested that it is essential to include bound phenolics in studies related to quantification and antioxidant activity evaluation of grains and cereals. However, the differences in bound phenolics and their relation to the antioxidant activity have not been well understood, partially due to genotypic diversity. Both DPPH and ABTS. + assays adopt a similar chemical reaction mechanism, in which a single electron transfer (SET assay) is involved in the reduction of a given compound. The addition of antioxidant compounds reduces ABTS. + or DPPH colored cations, thus causing a reagent decolorization that is measurable spectrophotometrically, depending on the antioxidant type, its concentration, and the incubation time (Brand-Williams et al., 1995) ABTS. + radical cation scavenging activity in the bran ranged from 19-24,11-16, and 9-19 μM TE/g in black, red, and NP rice, respectively. Among the black rice genotypes, the bran fraction of FCP and CP displayed higher ABTS. + activity of 24 and 21μM TE/g in the bound form. Red rice genotypes DMI and BKG displayed ABTS. + activity of 15 and 16 μM TE/g, respectively. NP rice genotypes PR126, PR114, and PR121 have ABTS. + activity of 19.0,14.8, and 12.9, μM TE/g in bound phenolics from bran fraction. In whole-grain fraction, black genotypes FCP and CP displayed higher ABTS. + activity of 8 and 12μM TE/g in bound form. Red rice genotypes BKG, DMI, KMA, and MSTY displayed ABTS. + activity of 3.1, 3.0, 2.9, and 2.8 μM TE/g in bound form in whole grain fraction. While for NP rice genotypes, PR 126 and BAS 370 displayed ABTS. + activity of 2.2 and 1.9 μM TE/g.

### Total anthocyanin, proanthocyanidin, and flavonoid content

Anthocyanins are colored, water-soluble pigments that belong to the phenolic group of compounds. The pigments usually exist in glycosylated forms. Anthocyanins are considered to be a flavonoid even though it has a positive charge at the oxygen atom of the C-ring of the basic flavonoid structure. It is also called the flavylium (2-phenylchromenylium) ion (Khoo, 2017). Total anthocyanin content in the bran was significantly higher than in the whole grain, suggesting that anthocyanins accumulated mainly in the bran layer (**Table 8**). Anthocyanin content in black rice bran fraction ranged from 1102-2364 mg/100g. In black rice genotypes, FCAP and CP displayed high content of anthocyanin, at 2364 and 2171 mg/100g, respectively. Red rice genotypes displayed much less content when compared to black rice and ranged from 22- 33 mg/100g. Among three different rice color groups, non-pigmented rice showed the least amount of anthocyanin content, ranging from 3.1-22 mg/100g. Some studies (Shao et al., 2014; Pang et al., 2018) stated that anthocyanin content was detected only in black rice. In the current study, we found low content of anthocyanin in both red and NP (**Table 8**). The whole grain fraction of black, red, and white rice displayed about 9-10-folds less content as compared to bran. The high anthocyanin content in the bran of black rice may contribute to their high phenolic content and high antioxidant capacity in bound form as compared to that in the free form of phenolics. Shao et al. (2014), reported that the anthocyanins of bran accounted for 97% of the total anthocyanins in the whole black grain, while those of embryo accounted for only 3%, suggesting that anthocyanins accumulated mainly in the bran layer. In the set of genotypes under the current study, anthocyanins of bran accounted for 89.6% of the total anthocyanins, which is close to the results reported by Shao et al. (2014). The major anthocyanin in rice was found to be cyanidin-3-glucoside. Abdel-Aal et al. (2006), This compound is reported to possess notable antioxidant and anti-inflammatory activities. Anthocyanins present in rice exhibit an antioxidant role in the biological cells by preventing cellular damage against harmful ultraviolet radiation.

Proanthocyanidins feature diverse phenolic hydroxyl groups, particularly ortho-dihydroxyl groups, and demonstrate the highest antioxidant capacity and free radical scavenging activity among numerous phenolic compounds (Buelga et al., 2022). Proanthocyanidins in the bran fraction ranged from 76-146 mg/100g in black rice, 694-2431mg/100g in red rice, and 1.9-3.5 mg/100g in NP rice (**Table 8**). The whole-grain fraction of three colored groups displayed about 8-10-folds less proanthocyanidin content than the bran fraction (**Table 8**). Among the three colored groups, red rice genotypes DMI, YKM, and BKG displayed higher content of proanthocyanidins (2431, 2048, and 1874 mg/100g, respectively). Cai et al. (2006) attributed the potent antioxidant activity shown in Sri Lankan traditional red rice varieties to the presence of proanthocyanidins. Red rice have been reported to possess significantly higher concentrations of proanthocyanidins compared to white and black rice (Hosoda et al., 2018; Shao et al., 2018). Finocchiaro et al. (2007), stated that the dominant phenolic antioxidant components in all traditional red-grained rice varieties were proanthocyanins but they did not detect anthocyanins in all the tested red-grained varieties. This statement was also supported by other researchers (Oki et al., 2002 and Reddy et al., 1995). Our findings show that both anthocyanin and proanthocyanidins were detected in black, red, and NP rice genotypes under the present study (**Table 8**). Flavonoids include a diverse group of polyphenolic secondary metabolites and have an essential role in regulating biological functions including antiviral, antibacterial, anti-inflammatory, antithrombotic, anti-allergic, and free radical scavenging properties (Shazia, 2013). The antioxidant activity of flavonoids is due to a combination of their iron chelating and free radical scavenging properties, as well as the inhibition of oxidases enzymes such as lipoxygenase, NADPH oxidase, cyclooxygenase, and xanthine oxidase. They then avoid the formation of reactive oxygen species and organic hydroperoxides (Escamilla et al., 2009), Moreover, flavonoids can inhibit enzymes by indirectly involving than in the oxidative process, such as phospholipase A2, and the same flavonoids can stimulate other enzymes with antioxidant activities like catalase and superoxide dismutase (Tan et al., 2018) Total flavonoid content in bran fraction ranging from 1187-1289 mg CE/100g in black rice, 745-1109 mg CE/100g in red rice, and 677-1028 mg CE/100g in NP rice was found in the present study. The black rice genotypes FCP and CP showed the highest value of total flavonoid content (TFC), at 1236 and1289 mg CE/100g in bran fraction (**Table 8**). The red rice genotypes DMI, BKG, and YKM displayed high content of flavonoids at 1022, 1109, and 970 mg CE/100g, respectively. NP rice genotypes Bas 386 and Pb Bas 7 also displayed high content of bran flavonoids (1001 and 1028 mg CE/100g respectively). Ti et al. (2014) reported the highest flavonoid content of 60.7 mg CE/100g DW in the polished rice fraction of variety ‘Y Liangyou’, while the bran fraction of the variety ‘Guinongzhan’ exhibited the highest flavonoid content (198.7 mg CE/100g). The variation in the content of flavonoids might be due to the influence of genetic and environmental factors that play an important role in the composition and content of phenolic compounds (Zhang et al., 2010).

### Phenolic acids and their relation to Total Flavonoid content (TFC) and antioxidant activity

The correlation between individual phenolic acid, TPC, and antioxidant activity in rice has not been widely studied. The correlation parameters used to assess the contribution of the individual phenolic acids to the antioxidant activity or TPC are shown in (**Supplementary Tables 1, 2**). Among black rice, whole grain free TPC had a significant positive correlation with VA, while SIA had a negative correlation (p ≤ 0.01). DPPH had a positive correlation with GA, VA, SYA, *p-*CA, and CH. *P*-HBA and SYA had a positive correlation with ABTS. +, while SIA had a negative correlation. Among red rice, whole grain free TPC had a positive correlation with SYA (**Supplementary Table 1**). Niu et al. (2013) showed that, in red rice, whole grain *p-*CA and VA may be responsible for antioxidant activity. In our study, we found that whole grain-free VA showed a significant positive correlation with TPC and DPPH in black and NP rice while *p*-CA displayed a significant positive correlation with DPPH activity in black and red rice (**Supplementary Table 1**). In red rice, DPPH activity had a negative correlation with 2,5 DHA and SIA. *p*- CA had a positive correlation. GA and VA had a positive correlation while *trans*-FA and CH had a negative correlation with ABTS+. Among NP whole grains, free TPC had a positive correlation with VA, whereas GA and VA had a positive correlation with DPPH. Pang et al. (2018), reported that there is a significant positive correlation among three parameters (TPC, DPPH, and ABTS. +) and GA, protocatechuic acid, 2,5-DHA, VA, FA, and SIA. Black rice whole grain bound TPC had a negative correlation with the flavonoids KAF and CH. DPPH had a positive correlation with 2,5-DHA and *trans-*FA, while *p*-HBA, SIA, VA, and CH had a negative correlation. VA had a positive correlation while *p*-CA and SIA had a negative correlation with ABTS. + antioxidant activity. Red rice whole grain bound TPC had a positive correlation with 2,5-DHA, *trans*-CA, and *trans*-FA, while VA, *p*-HBA, SIA, and CH had a negative correlation. Pang et al. (2018) observed that within red rice accessions, there was a negative correlation between the DPPH value and FA, while 2,5-DHA showed a positive correlation. In contrast, in our study, we found that *p*-HBA and SIA exhibited a significant negative correlation with three parameters: bound TPC, DPPH, and ABTS+ antioxidant activity (**Supplementary Table 1**) among red rice. However, Li et al. (2008) also found that bound TPC had a significantly negative correlation with SIA and isoferulic acid in winter wheat. Among NP rice accessions, whole grain bound TPC had a positive correlation with *p*-CA and SIA while *p*-HBA had a negative correlation.

In the bran fraction of black rice accessions, SIA and KAF had a significant positive correlation with free TPC and DPPH activity (**Supplementary Table 2**). 2, 5- DHA, SYA, and *t*-FA showed a significant positive correlation, while trans-CA showed a significant negative correlation with ABTS.+ antioxidant activity. In the bran fraction of red rice, *p*-CA had a significant positive correlation with free TPC, DDPH, and ABTS.+ antioxidant activity. In the bran fraction of NP rice, *p*-CA had a significant positive correlation with free TPC and DPPH activity while a significant negative correlation of TCA was obtained with free TPC and DPPH activity. SYA, CHA, and *t*-FA were positively correlated with ABTS.+ activity and may be responsible for the antioxidant activity of the free phenolics from bran (**Supplementary Table 2**). In the bran fraction of black rice, *p*-CA and SIA had a significant positive correlation with bound TPC and ABTS.+ activity. GA, VA, *t*-CA, and *t-FA* were positively correlated with the DPPH activity of the bound fraction. In the bran fraction of red rice accessions, 2,5-DHA exhibited a significant positive correlation with bound TPC and ABTS.+ antioxidant activity. CHA, *p*-CA, and SIA had a significant positive correlation with bound DPPH antioxidant activity. VA, SYA, *t*-FA, and SIA showed a significant negative correlation with bound TPC. In the bran fraction of NP rice, 2,5-DHA had a significant positive correlation with bound TPC and DPPH activity. GA, 2,5-DHA, *t*-CA, and *t*-FA contributed a negative correlation with bound TPC and DPPH antioxidant activity. Carlos et al. (2012) stated that the interactions among phenolic acids in a mixture can affect total DPPH antioxidant activity. It could therefore be surmised that individual phenolic acids present in free and bound forms in different fractions of the grain exert their antioxidant activities differently. They might act individually, synergistically, or antagonistically, and the molecular mechanisms need to be worked out in future studies. Thus, it can be inferred that the interactions among individual phenolic acids in an extract can also affect total antioxidant activities.

In conclusion, the individual phenolic acid composition and distribution of total anthocyanin, flavonoid, and proanthocyanidin content, total phenolics, and antioxidant capacity in the whole grain and bran of black, red, and NP rice were systematically investigated. Significant genotypic differences were observed in TPC and antioxidant capacity. The main bound phenolic acids in pigmented rice genotypes were p-HBA acid, p-CA, and t- FA, while in NP rice, these genotypes were p-HBA and t-FA. The main free phenolic acid was SYA in both pigmented and NP rices. 2,5-DHA was detected (mainly in bound form) for the first time in NP rice genotypes; the levels of bound TPC were significantly higher than free TPC in both the whole grain and bran fraction in pigmented and NP rice genotypes. The black and red rice genotypes exhibited higher TPC, TFC, and antioxidant activities compared to NP rice. A significant level of TFC was observed in NP rice, especially Basmati genotypes. The study contributes toward further understanding of the distribution of individual free and bound phenolic acids in pigmented and NP rice in whole grain and bran fractions. It may assist rice breeders and, eventually, farmers with new opportunities to promote the production of rice with enhanced levels of phytochemicals. This research validated the use of rice bran as a rich source of phytochemicals with high antioxidant capacities.

## Data availability statement

The original contributions presented in the study are included in the article/**Supplementary Material**. Further inquiries can be directed to the corresponding author.

## Author contributions

GA: Writing – original draft. NS: Writing – review & editing, Supervision, Project administration, Methodology, Investigation, Conceptualization. KP: Visualization, Software, Writing – review & editing, Formal analysis, Data curation. RK: Writing – review & editing, Resources. GS: Writing – review & editing, Software, Formal analysis, Data curation. NP: Writing – review & editing, Formal analysis, Data curation. TT: Writing – review & editing, Visualization, Validation, Data curation.

## Supplementary material

Supplementary Table 1 Pearson pair-wise correlations between individual phenolic acid, total phenolic content and antioxidant activity of free and bound phenolics of whole grain.

Supplementary Table 2 Pearson pair-wise correlations between individual phenolic acid, total phenolic content and antioxidant activity of free and bound phenolics of Bran.

## References

[B1] Abdel-Aal E. S. M. Young J. C. Rabalski I. (2006). Anthocyanin’s composition in black, blue, pink, purple, and red cereal grains. J. Agric. Food Chem. 54, 4696–4704. doi: 10.1021/jf0606609 16787017

[B2] Adom K. K. Liu R. H. (2002). Antioxidant activity of grains. J. Agric. Food Chem. 50, 6182–6187. doi: 10.1021/jf0205099 12358499

[B3] Alessandra C. P. Daniel G. Neiva D. R. (2016). Extraction of anthocyanins and polyphenols from black rice (*Oryza sativa* L.) by modeling and assessing their reversibility and stability. Food Chem. 191, 12–20. doi: 10.1016/j.foodchem.2015.02.045 26258696

[B4] Alves G. H. Cristiano D. F. Patrícia G. V. Jander L. F. M. Moacir C. E. Nathan L. V.. (2016). The revisited levels of free and bound phenolics in rice: Effects of the extraction procedure. Food Chem. doi: 10.1016/j.foodchem.2016.03.107 27132831

[B5] Bagchi B. T. Krishnendu C. Sivashankari M. Sankhajit R. Awadhesh K. Tufleuddin B.. (2021). Effect of different processing technologies on phenolic acids, flavonoids and other antioxidants content in pigmented rice. J. Cereal Sci. 100, 103–263. doi: 10.1016/j.jcs.2021.103263

[B6] Bani C. Di L. C. Restani P. M. F. Colombo F. (2023). Phenolic profile and *in vitro* antioxidant activity of different corn and rice varieties. Plants 12, 448. doi: 10.3390/plants12030448 36771533 PMC9920881

[B7] Brand-Williams W. Cuvelier M. E. Berset C. L. W. T. (1995). Use of a free radical method to evaluate antioxidant activity. LWT - Food Sci. Technol. 28, 25–30. doi: 10.1016/S0023-6438(95)80008-5

[B8] Buelga C. S. Manzano S. G. Ana M. Paramás G. (2022). White wine polyphenols and health. White Wine Technol. 71, 205–220. doi: 10.3390/molecules26185537

[B9] Cai Y. Z. Sun M. Xing J. Luo Q. Corke H. (2006). Structure-radical scavenging activity relationships of natural phenolic compounds from traditional Chinese medicinal plants. Life Sci. 78, 2872–2888. doi: 10.1016/j.lfs.2005.11.004 16325868

[B10] Carlos P. Chávez H. G. Mundo J. S. Namiesnik J. R. R. Gorinstein S. Aguilar G. A. (2012). Antioxidant interactions between major phenolic compounds found in ‘Ataulfo’ mango pulp: Chlorogenic, gallic, protocatechuic and vanillic acids. Molecules 17, 12657–12664. doi: 10.3390/molecules171112657 23103532 PMC6268240

[B11] Chaiyasut C. Sivamaruthi B. Pengkumsri N. Sirilun S. Peerajan S. Chaiyasut K.. (2015). Anthocyanin profile and its antioxidant activity of widely used fruits, vegetables, and flowers in Thailand. J. Food Drug Anal. 9, 218–224. doi: 10.22159/ajpcr.2016.v9i6.14245

[B12] Das A. Golder A. K. Das C. (2016). Enhanced extraction of rebaudioside-A: Experimental, response surface optimization and prediction using artificial neural network. Ind. Crops Prod. 65, 415–421. doi: 10.1016/j.indcrop.2014.11.006

[B13] Dewanto V. Wu X. Adom K. K. Liu R. H. (2002). Thermal processing enhances the nutritional value of tomatoes by increasing total antioxidant activity. J. Agric. Food Chem. 8, 50(10):3010–4. doi: 10.1021/jf0115589 11982434

[B14] Escamilla C. O. Cuevas E. Y. Guevara J. (2009). Flavonoids y sus acciones antioxidantes. Rev. Fac Cienc Med. Cordoba 52, 73–75.

[B15] Finocchiaro F. Ferrari B. Gianinetti A. (2010). A study of biodiversity of flavonoid content in the rice caryopsis evidencing simultaneous accumulation of anthocyanins and proanthocyanidins in a black-grained genotype. J. Cereal Sci. 51, 28–34. doi: 10.1016/j.jcs.2009.09.003

[B16] Finocchiaro F. Ferrari B. Gianinetti A. Dall’Asta C. Galaverna G. Scazzina F.. (2007). Characterization of antioxidant compounds of red and white rice and changes in total antioxidant capacity during processing. Mol. Nutr. Food Res. 51, 1006–1019. doi: 10.1002/mnfr.200700011 17639995

[B17] Gong E. S. Luo S. J. Li T. Liu C. M. Zhang W. G. Chen J.. (2017). Phytochemical profiles and antioxidant activity of brown rice varieties. Food Chem. 227, 432–443. doi: 10.1016/j.foodchem.2017.01.093 28274454

[B18] Hosoda K. Sasahara H. Matsushita K. Tamura Y. Miyaji M. Matsuyama H. (2018). Anthocyanin and proanthocyanidin contents, antioxidant activity, and in *situ* degradability of black and red rice grains. Asian-Australias J. Anim. Sci. 31, 1213–1220. doi: 10.5713/ajas.17.0655 PMC6043450 29514441

[B19] Huang S. H. Ng L. T. (2012). Quantification of polyphenolic content and bioactive constituents of some commercial rice varieties in Taiwan. J. Food Compost Anal. 26, 122–127. doi: 10.1016/j.jfca.2012.03.009

[B20] Huang Y. P. Lai H. M. (2016). Bioactive compounds and antioxidative activity of colored rice bran. J. Food Drug Anal. 24, 564–574. doi: 10.1016/j.jfda.2016.01.004 28911562 PMC9336675

[B21] Iqbal S. Bhanger M. I. Anwar F. (2005). Antioxidant properties and components of some commercially available varieties of rice bran in Pakistan. Food Chem. 93, 265–272. doi: 10.1016/j.foodchem.2004.09.024

[B22] Irakli M. N. Samanidou V. F. Biliaderis C. G. Papadoyannis I. N. (2012). Simultaneous determination of phenolic acids and flavonoids in rice using solid-phase extraction and RP-HPLC with photodiode array detection. J. Sep Sci. 35, 1603–1611. doi: 10.1002/jssc.201200140 22761138

[B23] Kaur P. Singh N. Pal P. Kaur A. (2018). Variation in composition, protein and pasting characteristics of different pigmented and non-pigmented rice (*Oryza sativa* L.) grown in Indian Himalayan region. J. Food Sci. Technol. 55, 3809–3820. doi: 10.1007/s13197-018-3361-1 30150841 PMC6098773

[B24] Khoo H. C. (2017). Anthocyanidins and anthocyanins: colored pigments as food, pharmaceutical ingredients, and the potential health benefits. Food Nutr. Res. 5, 12–15. doi: 10.1080/16546628.2017.1361779 PMC5613902 28970777

[B25] Kunnam J. Pinta W. Ruttanaprasert R. Bunphan D. Thabthimtho T. Aninbon C. (2023). Stability of phenols, antioxidant capacity and grain yield of six rice genotypes. Plants. 12, 2787. doi: 10.3390/plants12152787 37570941 PMC10421503

[B26] Li L. Shewry P. R. Ward J. L. (2008). Phenolic acids in wheat varieties in the health grain diversity screen. J. Agri Food Chem. 56, 9732–9739. doi: 10.1021/jf801069s 18921977

[B27] Liyana-Pathirana C. M. Shahidi F. (2006). Importance of insoluble-bound phenolics to antioxidant properties of wheat. J. Agric. Food Chem. 54, 1256–1264. doi: 10.1021/jf052556h 16478245

[B28] Min B. Gu L. Mcclung A. M. Bergman C. J. Chen M. (2012). Free and bound total phenolic concentrations, antioxidant capacities and profiles of proanthocyanidins and anthocyanins in whole grain rice (*Oryza sativa* L.) of different bran colours. Food Chem. 133, 715–722. doi: 10.1016/j.foodchem.2012.01.079

[B29] Min B. McClung A. M. Chen M. H. (2011). Phytochemicals and antioxidant capacities in rice brans of different color. J. Food Sci. 76, 117–126. doi: 10.1111/j.1750-3841.2010.01929.x 21535639

[B30] Mira H. Bae J. S. Ban J. J. Shin H. S. Lee D. H. Chung J. H. (2009). Black rice (*Oryza sativa* L.) extract modulates ultraviolet-induced expression of matrix metalloproteinases and procollagen in a skin cell model. Int. J. Mol. Med. 41, 3073–3080. doi: 10.3892/ijmm.2018.3508 29484380

[B31] Miyazawa A. Fujiyoshi Y. Unwin N. (2003). Structure and gating mechanism of the acetylcholine receptor pore. Nature 423, 949–955. doi: 10.1038/nature01748 12827192

[B32] Naczk M. Shahidi F. (2006). Phenolics in cereals, fruits and vegetables: occurrence, extraction and analysis. J. Pharm. BioMed. 41, 1523–1542. doi: 10.1016/j.jpba.2006.04.002 16753277

[B33] Niu Y. Gao B. Slavin M. Zhang X. Yang F. Bao J.. (2013). Phytochemical compositions, and antioxidant and anti-inflammatory properties of twenty-two red rice samples grown in Zhejiang. Food Sci. Tech. 54, 521–527. doi: 10.1016/j.lwt.2013.06.018

[B34] Oki T. Masuda M. Kobayashi M. Nishida Y. Furuta S. Suda I.. (2002). Polymeric procyanidins as radical- scavenging components in red-hulled rice. J. Agric. Food Chem. 50, 7524–7529. doi: 10.1021/jf025841z 12475265

[B35] Paiva F. F. Vanier N. L. Berrios J. D. J. Pinto V. Z. Wood D. Williams T. (2016). Polishing and parboiling effect on the nutritional and technological properties of pigmented rice. Food Chem. 191, 105–112. doi: 10.1016/j.foodchem.2015.02.047 26258708

[B36] Pang Y. Sulaiman A. Yanjie X. Trust B. Zhiwei Z. Yafang S.. (2018). Bound phenolic compounds and antioxidant properties of whole grain and bran of white, red and black rice. Food Chem. 240, 212–221. doi: 10.1016/j.foodchem.2017.07.095 28946264

[B37] Praveen S. Memon S. Q. Siyal A. N. Memon N. Khuhawar M. Y. (2016). Large sample staking of rice polyphenols prior to their determination by nonaqueous capillary electrophoresis. Food Anal. Methods 9, 2152–2160. doi: 10.1007/s12161-015-0394-1

[B38] Qiu Y. Liu Q. Beta T. (2010). Antioxidant properties of commercial wild rice and analysis of soluble and insoluble phenolic acids. Food Chem. 121, 140–147. doi: 10.1016/j.foodchem.2009.12.021

[B39] Reddy V. S. Dash S. Reddy A. R. (1995). Anthocyanin pathway in rice (*Oryza sativa* L.): identification of a mutant showing dominant inhibition of anthocyanins in leaf and accumulation of proanthocyanidins in pericarp. Theor. Appl. Genet. 91, 301–312. doi: 10.1007/BF00220892 24169778

[B40] Saura F. C. Serrano J. Goni I. (2007). Intake and bio accessibility of total polyphenols in a whole diet. Food Chem. 101, 492–501. doi: 10.1016/j.foodchem.2006.02.006

[B41] Shao Y. Bao J. (2015). Polyphenols in whole rice grain: Genetic diversity and health benefits. Food Chem. 180, 86–97. doi: 10.1016/j.foodchem.2015.02.027 25766805

[B42] Shao Y. Hu Z. Y. Mou R. Zhu Z. Beta T. (2018). Phenolic acids, anthocyanins, proanthocyanidins, antioxidant activity, minerals and their correlations in non-pigmented, red, and black rice. Food Chem. 239, 733–741. doi: 10.1016/j.foodchem.2017.07.009 28873629

[B43] Shao Y. F. Xu F. F. Sun X. Bao J. S. Beta T. (2014). Identification and quantification of phenolic acids and anthocyanins as antioxidants in bran, embryo and endosperm of white, red and black rice kernels (*Oryza sativa* L.). J. Cereal Sci. 59, 211–218. doi: 10.1016/j.jcs.2014.01.004

[B44] Shao Y. Xu F. Sun X. Bao J. Beta T. (2015). Phenolic acids, anthocyanins, and antioxidant capacity in rice (*Oryza sativa* L.) grains at four stages of development after flowering. Food Chem. 143, 90–96. doi: 10.1016/j.foodchem.2013.07.042 24054217

[B45] Sharmila K. Sudarshana S. Ranjith M. Ruwan R. Gevindu W. (2023). Development and validation of a method based on liquid chromatography-mass spectrometry for comprehensive profiling of phenolic compounds in rice. Microchemical J. 193, 109211. doi: 10.1016/j.microc.2023.109211

[B46] Shazia U. (2013). Screening for antioxidant and free radical scavenging potential of extracts of leaves and flowers of Calotropis gigantea. Asian J. Pharm. Clin. Res. 6, 97–100.

[B47] Shen Y. Jin L. Xiao P. Lu Y. Bao J. S. (2009). Total phenolics, flavonoids, antioxidant capacity in rice grain and their relations to grain color, size, and weight. J. Cereal Sci. 49, 106–111. doi: 10.1016/j.jcs.2008.07.010

[B48] Siva R. Kumar K. Rajasekaran C. (2013). Genetic diversity study of important Indian rice genotypes using biochemical and molecular markers. Afr. J. Biotech. 12, 1004–1009.

[B49] Sumczynski D. Kotásková E. Druzˇbıková H. Mlcek J. (2015). Determination of contents and antioxidant activity of free and bound phenolics compounds and *in vitro* digestibility of commercial black and red rice (*Oryza* sativa L.) varieties. Food Chem. 211. doi: 10.1016/j.foodchem.2016.05.081 27283641

[B50] Sun B. Ricardo da Silva J. M. Spranger I. (1998). Critical factors of vanillin assay for catechins and proanthocyanidins. J. Agri Food Chem. 46, 4267–4274. doi: 10.1021/jf980366j

[B51] Tan B. L. Mohd E. N. Winnie P. P. L. Heshu S. R. (2018). Antioxidant and oxidative stress: A mutual interplay in age-related diseases. Front. Pharma. 9. doi: 10.3389/fphar.2018.01162 PMC6204759 30405405

[B52] Ti H. Li Q. Zhang R. Zhang M. Deng Y. Wei Z. (2014). Free and bound phenolic profiles and antioxidant activity of milled fractions of different indica rice varieties cultivated in southern China. Food Chem. 159, 166–174. doi: 10.1016/j.foodchem.2014.03.029 24767040

[B53] Verardo V. Gómez-Caravaca A. M. Marconi E. Segura-Carretero A. Garrido-Frenich A. Fernández-Gutiérrez. (2016). Determination of lipophilic and hydrophilic bioactive compounds in raw and parboiled rice bran. R S C Adv. 6, 50786–50796. doi: 10.1039/C6RA04836F

[B54] Yao Y. Sang W. Zhou M. Ren G. (2010). Antioxidant and α-glucosidase inhibitory activity of colored grains in China. J. Agric. Food Chem. 58, 770–774. doi: 10.1021/jf903234c 19904935

[B55] Zaupa M. Del Calani L. Rio D. Brighenti F. Pellegrini N. (2015). Characterization of total antioxidant capacity and (poly) phenolic compounds of differently pigmented rice varieties and their changes during domestic cooking. Food Chem. 187, 338–347. doi: 10.1016/j.foodchem.2015.04.055 25977035

[B56] Zhang H. Shao Y. Bao J. Beta T. (2015). Phenolic compounds and antioxidant properties of breeding lines between the white and black rice. Food Chem. 172, 630–639. doi: 10.1016/j.foodchem.2014.09.118 25442600

[B57] Zhang M. W. Zhang R. F. Zhang F. X. Liu R. H. (2010). Phenolic profiles and antioxidant activity of black rice bran of different commercially available varieties. J. Agric. Food Chem. 58, 7580–7587. doi: 10.1021/jf1007665 20521821

[B58] Zhou Z. Robards K. Helliwell S. Blanchard C. (2004). The distribution of phenolic acids in rice. Food Chem. 87, 401–406. doi: 10.1016/j.foodchem.2003.12.015

