# Supplementary material for: Unveiling the distribution of free and bound phenolic acids, flavonoids, anthocyanins, and proanthocyanidins in pigmented and non-pigmented rice genotypes
Source: Front Plant Sci. 2024 Apr 10;15:1324825. doi: 10.3389/fpls.2024.1324825 (PMC11039891; doi:10.3389/fpls.2024.1324825)
Supplement: Supplementary Table 1 — Pearson pair-wise correlations between individual phenolic acid, total phenolic content and antioxidant activity of free and bound phenolics of whole grain. [file Table_1.docx]

Supplementary Table 1. Pearson pair-wise correlations between individual phenolic acid, total phenolic content and antioxidant activity of free and bound phenolics of whole grain

|  |  | **Black rices** |  |  | **Red rices** |  |  | **Non- pigmented rices** |  |
| --- | --- | --- | --- | --- | --- | --- | --- | --- | --- |
| **Free phenolics** | **TPC** | **DPPH** | **ABTS** | **TPC** | **DPPH** | **ABTS** | **TPC** | **DPPH** | **ABTS** |
| **GA** | -0.2 | 0.757** | -0.345 | -0.308 | -0.088 | 0.634** | 0.146 | 0.808** | -0.676** |
| **2,5-DHA** | -0.131 | -0.445 | -0.189 | 0.901** | -0.712** | -0.41 | 0.09 | nd | 0.064 |
| **VA** | 0.698** | 0.809** | -0.002 | -0.623** | 0.285 | 0.743** | 0.484* | 0.928** | -0.369 |
| ***p*-HBA** | 0.336 | 0.071 | 0.854** | 0.268 | -0.462 | 0.016 | -0.507 | 0.058 | -0.915** |
| **SYA** | 0.121 | 0.523** | 0.648** | 0.573* | -0.213 | -0.732** | -0.072 | -0.614 | -0.436 |
| **CHA** | -0.004 | -0.4 | 0.178 | 0.015 | 0.338 | -0.413 | -0.331 | -0.571 | 0.456 |
| ***t-*CA** | -0.163 | -0.094 | -0.753 | 0.239 | -0.054 | -0.375 | 0.346 | -0.317 | -0.614 |
| ***t-*FA** | 0.311 | -0.422 | -0.368 | 0.255 | 0.126 | -0.652** | -0.093 | -0.631 | -0.111 |
| ***p*-CA** | 0.273 | 0.698** | -0.049 | -0.667** | 0.620** | 0.342 | 0.303 | 0.599 | -0.283 |
| **SIA** | -0.598** | 0.059 | -0.899** | 0.402 | -0.658** | 0.162 | 0.212 | 0.019 | -0.915** |
| **KAF** | 0.391 | 0.364 | -0.335 | -0.393 | 0.500** | -0.004 | -0.212 | 0.186 | -0.402 |
| **CH** | 0.358 | 0.622** | -0.124 | -0.472* | 0.366 | -0.415* | -0.401 | 0.455 | -0.243 |
| **Bound phenolics** |  |  |  |  |  |  |  |  |  |
| **GA** | -0.121 | 0.406 | 0.471 | -0.504 | 0.221 | 0.716 | -0.011 | 0.511 | -0.21 |
| **2,5-DHA** | 0.181 | 0.655** | -0.221 | 0.969** | 0.589* | 0.255 | -0.033 | -0.221 | -0.076 |
| **VA** | -0.194 | -0.486* | 0.521* | -0.583* | -0.340 | 0.290 | -0.219 | 0.463 | -0.070 |
| ***p*-HBA** | -0.037 | -0.511* | -0.461 | -0.625** | -0.514* | -0.671** | -0.598** | -0.438 | 0.335 |
| **SYA** | 0.069 | -0.462 | 0.105 | -0.109 | -0.158 | 0.137 | -0.329 | -0.084 | 0.312 |
| **CHA** | -0.161 | 0.426 | -0.344 | 0.463 | 0.229 | 0.052 | 0.445 | -0.182 | -0.131 |
| ***t-*CA** | -0.435 | 0.090 | -0.324 | 0.503* | 0.363 | -0.107 | 0.034 | -0.208 | 0.245 |
| ***t-*FA** | 0.332 | 0.692** | -0.257 | 0.985** | 0.456 | 0.471* | -0.093 | -0.290 | -0.195 |
| ***p*-CA** | -0.244 | 0.157 | -0.538* | 0.296 | 0.058 | -0.151 | 0.930** | -0.386 | 0.105 |
| **SIA** | -0.229 | -0.719** | -0.477* | -0.670** | -0.682** | -0.423 | 0.645** | -0.451 | 0.358 |
| **KAF** | -0.542* | -0.375 | 0.337 | -0.139 | -0.046 | 0.364 | -0.003 | 0.368 | 0.052 |
| **CH** | -0.498* | -0.658** | 0.131 | -0.630** | 0.019 | -0.915** | -0.143 | 0.162 | 0.622** |

*Indicate significant at P≤ 0.005, ** indicate significant at P≤ 0.001.

GA, gallic acid: 2,5-DHA, 2,5-dihydroxybenzoic acid; VA, vanillic acid; *p*-HBA, p hydroxybenzoic acid; SYA, syringic acid; CHA, Chlorogenic acid; *t-*CA, *trans-*cinnamic acid; *t-*FA, *trans-*ferulic acid; *p*-CA, *p*-coumaric acid; SIA, sinapic acid; KAF, kaempferol; CH, catechin hydrat
