# Supplementary material for: Unveiling the distribution of free and bound phenolic acids, flavonoids, anthocyanins, and proanthocyanidins in pigmented and non-pigmented rice genotypes
Source: Front Plant Sci. 2024 Apr 10;15:1324825. doi: 10.3389/fpls.2024.1324825 (PMC11039891; doi:10.3389/fpls.2024.1324825)
Supplement: Supplementary Table 2 — Pearson pair-wise correlations between individual phenolic acid, total phenolic content and antioxidant activity of free and bound phenolics of Bran. [file Table_2.docx]

Supplementary Table2. Pearson pair-wise correlations between individual phenolic acid, total phenolic content and antioxidant activity of free and bound phenolics of bran

|  |  | **Black rices** |  |  | **Red rices** |  |  | **Non- pigmented rices** |  |
| --- | --- | --- | --- | --- | --- | --- | --- | --- | --- |
| **Free phenolics** | **TPC** | **DPPH** | **ABTS** | **TPC** | **DPPH** | **ABTS** | **TPC** | **DPPH** | **ABTS** |
| **GA** | 0.179 | 0.778* | -0.625 | 0.243 | -0.936 | 0.179 | -0.485 | -0.936** | 0.028 |
| **2,5DHA** | 0.544 | 0.498 | 0.696* | -0.942** | 0.028 | 0.544 | 0.607* | 0.23 | -0.451 |
| **VA** | 0.362 | 0.912** | -0.363 | -0.113 | 0.917** | 0.362 | -0.188 | -0.792* | 0.354 |
| **p-HBA** | 0.114 | 0.537 | 0.201 | -0.430 | 0.179 | 0.114 | 0.778** | -0.05 | 0.544 |
| **SYA** | 0.194 | -0.158 | 0.974** | -0.888** | -0.544 | 0.194 | -0.596** | 0.753* | 0.753* |
| **CHA** | 0.513 | 0.630 | 0.477 | -0.837** | 0.238 | 0.513 | -0.406 | -0.03 | 0.895** |
| ***t-*CA** | 0.024 | 0.553 | -0.899** | 0.616 | 0.886** | 0.024 | -0.671** | -0.962** | -0.399 |
| ***t-*FA** | 0.596 | 0.151 | 0.891** | -0.928** | -0.316 | 0.596 | 0.3495 | 0.56 | 0.872** |
| ***p*-CA** | 0.379 | 0.211 | -0.018 | 0.870** | 0.654** | 0.379 | 0.598** | 0.900** | -0.470* |
| **SIA** | 0.723** | 0.870** | 0.016 | 0.032 | 0.379 | 0.723** | 0.0867 | 0.962** | -0.199 |
| **KAF** | 0.592** | 0.900** | 0.123 | 0.962** | 0.572** | 0.592** | -0.096 | 0.088* | -0.116 |
| **CH** | -0.231 | 0.580** | 0.167 | 0.337 | 0.341 | -0.231 | 0.530* | 0.33 | -0.620** |
| **Bound phenolics** |  |  |  |  |  |  |  |  |  |
| **GA** | -0.194 | 0.723** | -0.071 | 0-.152 | 0.269 | 0.178 | 0.702** | 0.246 | -0.495 |
| **2,5DHA** | -0.033 | 0.307 | -0.125 | 0.828** | -0.195 | 0.471* | 0.969** | 0.534* | -0.301 |
| **VA** | -0.219 | 0.608** | 0.289 | -0.922** | 0.154 | 0.088 | -0.583* | 0.276 | -0.500* |
| **p-HBA** | -0.598** | -0.414 | -0.331 | -0.468 | -0.511* | -0.554* | -0.625** | -0.671** | 0.228 |
| **SYA** | -0.329 | 0.176 | -0.275 | -0.645** | 0.147 | -0.383 | -0.109 | -0.411 | 0.012 |
| **CHA** | 0.445 | -0.263 | 0.702** | -0.373 | 0.594** | -0.472 | 0.463 | -0.327 | 0.343 |
| ***t-*CA** | 0.034 | 0.707** | 0.136 | 0.760** | 0.530 | 0.096 | 0.503* | 0.269 | -0.381 |
| ***t-*FA** | -0.093 | 0.650** | 0.167 | -0.679** | 0.451 | -0.020 | 0.985** | 0.122 | -0.379 |
| ***p*-CA** | 0.930** | -0.355 | 0.605** | -0.163 | 0.687** | -0.073 | 0.296 | 0.107 | 0.534* |
| **SIA** | 0.645** | -0.610** | 0.548* | 0.000 | 0.567* | -0.568* | -0.670** | -0.394 | 0.740** |
| **KAF** | -0.003 | 0.121 | 0.016 | -0.657** | 0.451 | -0.430 | -0.139 | -0.352 | 0.157 |
| **CH** | -0.143 | -0.658** | 0.131 | 0.358 | 0.019 | -0.915** | -0.630** | -0.899** | 0.622** |

*Indicate significant at P≤ 0.005, ** indicate significant at P≤ 0.001.GA, gallic acid: 2,5-DHA, 2,5-dihydroxybenzoic acid; VA, vanillic acid; *p*-HBA, p hydroxybenzoic acid; SYA, syringic acid;CHA, Chlorogenic acid; *t-*CA, *trans-*cinnamic acid; *t-*FA, *trans-*ferulic acid; *p*-CA, *p*-coumaric acid; SIA, sinapic acid; KAF, kaempferol; CH, catechin
